# Comparative transcriptome and metabolome profiling unveil genotype-specific strategies for drought tolerance in cotton

**DOI:** 10.3389/fpls.2025.1610552

**Published:** 2025-06-13

**Authors:** Aixia Han, Wanwan Fu, Yunhao Liusui, Xingyue Zhong, Xin Zhang, Ziyu Wang, Yuanxin Li, Jingbo Zhang, Yanjun Guo

**Affiliations:** Xinjiang Key Laboratory of Special Species Conservation and Regulatory Biology, College of Life Science, Xinjiang Normal University, Urumqi, China

**Keywords:** cotton, drought-resistant varieties, transcriptome, metabolome, hub gene

## Abstract

As a globally important economic crop, cotton often faces yield and quality limitations due to drought stress. To elucidate drought tolerance mechanisms, this study screened a drought-tolerant variety (64-22-3) and a drought-sensitive variety (Anmian 3, A3) from five drought-resistant and five drought-sensitive materials, respectively. Integrated transcriptomic and metabolomic analyses revealed 7,351 differentially expressed genes (DEGs) in the drought-tolerant variety under drought treatment (5,034 upregulated, 2,317 downregulated), while the drought-sensitive variety exhibited 5,009 DEGs (3,222 upregulated, 1,787 downregulated). Metabolomic profiling identified 169 differentially accumulated metabolites (DAMs) (120 upregulated, 49 downregulated) in 64-22–3 and 173 DAMs (120 upregulated, 53 downregulated) in A3. KEGG enrichment analysis showed that DEGs and DAMs in both varieties were significantly enriched in secondary metabolite biosynthesis, flavonoid biosynthesis, and sesquiterpenoid/triterpenoid biosynthesis. Notably, the drought-tolerant variety displayed specific enrichment in phenylpropanoid biosynthesis, linoleic acid metabolism, and glucosinolate biosynthesis, suggesting their roles in drought adaptation. Weighted gene co-expression network analysis (WGCNA) of 2,064 unique DEGs and 20 key metabolites in the drought-tolerant variety identified blue and turquoise modules as strongly associated with metabolite accumulation, with core hub genes Ghi_D06G05631 and Ghi_A13G12271, which encode TOPLESS-related 1 protein and CIPK6 (CBL-interacting protein kinase 6) separately. Transcription factor (TF) analysis revealed seven high-connectivity TF families (HSF, Golden2-like, SNF2, mTERF, bHLH, C2H2, B3) in the blue module and six TF families (Tify, ARR-B, AUX/IAA, bHLH, Alfin-like, LUG) in the turquoise module, suggesting their coordinated regulation of drought responses. This study systematically elucidates the molecular network underlying cotton’s drought adaptation, providing critical insights for identifying key drought-resistant genes and developing resilient cultivars.

## 1 Introduction

Cotton is one of the most economically significant natural fiber crops globally (Geng et al., 2024). As the largest producer and consumer of cotton globally, China faces significant economic implications from potential shortages of cotton and cotton-derived products. Current cultivation practices concentrate cotton production in arid and semi-arid zones, rendering the crop highly susceptible to drought stress. Emerging evidence underscores drought as the primary abiotic constraint that impairs cotton growth, development, yield formation, and fiber quality (Geng et al., 2024). Addressing this challenge necessitates a systematic exploration of drought response mechanisms at the molecular level. By identifying candidate drought-resistant genes, characterizing key regulatory networks, and implementing marker-assisted breeding strategies, researchers can develop stress-tolerant cotton cultivars. This integrative approach holds promise for alleviating genetic resource limitations and optimizing water use efficiency in cotton production systems (Yin et al., 2024a).

Current research has revealed that plants have evolved sophisticated multi-level response mechanisms to cope with drought stress, which can be systematically categorized as follows: (1) Rapidly initiating stomatal closure and stress-related gene expression via the ABA pathway (Yoshida et al., 2014); (2) Synthesizing osmoprotectants like proline and betaine for cellular turgor maintenance (Hennig, 2012); (3) Activating the antioxidant enzyme system, including superoxide dismutase (SOD) and catalase (CAT), to scavenge reactive oxygen species (ROS) (Ali et al., 2022); (4) Accumulating secondary metabolites such as flavonoids and terpenoids to enhance cell membrane stability (Jan et al., 2024). Notwithstanding these conserved adaptive strategies, drought responses involve intricate molecular networks, signaling cascades, and metabolic reprogramming that exhibit substantial interspecific and intraspecific variations. Therefore, gaining mechanistic insights into species-specific or genotype-specific drought responses is critical for precise identification of key regulatory genes and development of targeted breeding strategies.

Transcriptomics is the study of the overall gene expression in a biological organism under specific conditions. It reveals the molecular mechanisms of gene regulatory networks and biological processes by analyzing the types and quantities of RNA molecules, such as mRNA, lncRNA, and miRNA. Recent advances in high-throughput sequencing technologies have revolutionized plant transcriptomics, enabling comprehensive investigations into growth regulation, stress adaptation, and metabolic networks (Yin et al., 2023). At the same time, the advancement of mass spectrometry technology has also promoted the widespread application of metabolomics in plant research. Metabolomics is a technology for the quantitative analysis of all metabolites in a biological organism. It can detect small molecules and exogenous substances, including endogenous substances in tissues or organs with a relative molecular mass usually less than 1000. Metabolites represent the final products of cellular regulatory processes, serving as the ultimate indicators of biological systems’ responses to genetic or environmental changes (Liang et al., 2022).

During the last few years, advancements in high-throughput sequencing technologies have driven a paradigmatic shift in plant drought research. Integrated transcriptomic and metabolomic analysis offers a systematic approach to elucideate cooperative networks between gene expression regulation and metabolite accumulation. Firstly, transcriptomic data provide genetic-level explanations for metabolite biosynthesis, transport, and regulation. For instance, transcriptomic profiling may identify upregulated expression of key enzyme-encoding genes involved in carbohydrate metabolism, which can be correlated with corresponding metabolite changes observed in metabolomic datasets. This establishes direct links between gene expression dynamics and metabolic reprogramming. Moreover, metabolomic data can corroborate transcriptomic findings by functional validation. When transcriptomics identifies DEGs under drought whose functions remain elusive, metabolomic analysis may reveal specific metabolite perturbations associated with these genes, enabling hypotheses regarding their roles in metabolic pathway regulation.

Integrated transcriptomic and metabolomic analyses have revealed key molecular responses to drought in switchgrass. The drought-tolerant *Alamo* genotype upregulates diterpenoid biosynthetic genes, leading to root diterpenoid accumulation and enhanced stress tolerance (Tiedge et al., 2022). Metabolomic profiling demonstrated that both drought and cold stress stimulate raffinose, trehalose-6-phosphate, proline and monosaccharide accumulation, coupled with increased expression of corresponding biosynthetic genes (Guo et al., 2021). Correlation analysis between transcriptomics and metabolomics has revealed that *LOC110713661* and *LOC110738152* may be key genes for drought tolerance in quinoa, with DEGs and metabolites being annotated to starch and sucrose metabolism as well as flavonoid biosynthesis pathways, indicating that these metabolic pathways are crucial for enhancing quinoa’s drought tolerance (Huan et al., 2022). Integrated transcriptomic and metabolomic analyses demonstrated that *ZmGLK44* directly binds to and activates tryptophan synthase TSB2, regulating the tryptophan biosynthesis pathway in maize and playing a key role in metabolic regulation and drought response (Zhang et al., 2021). Transcriptomic and metabolomic profiling demonstrated that sweet potato boosts drought resistance through the activation of antioxidant-related genes and increased flavonoid production under drought stress (Yin et al., 2024b). Studies demonstrate that proline, tryptophan, and phenylalanine serve as crucial amino acids for maize drought adaptation, with enhanced expression of tryptophan biosynthesis genes (*ZmAO1*, *ZmCAT1*, and *ZmYUC6*) significantly contributing to drought resistance regulation (Li et al., 2024b).

WGCNA has emerged as an effective tool for identifying functional genes by linking gene expression data with phenotypic traits, constructing gene coexpression networks, and identifying gene modules and hub genes closely associated with plant traits (Ma et al., 2024a). Through WGCNA analysis of transcriptomic data, the hub gene *GhNAC072* was identified, and subsequent functional studies revealed that silencing *GhNAC072* reduced cotton’s tolerance to drought stress, while overexpressing this gene in *Arabidopsis* enhanced its drought tolerance (Mehari et al., 2021). WGCNA analysis identified 851 core drought-resistant genes in XZSN (the drought-resistant genotype of Tartary buckwheat), most of which were induced earlier and more rapidly by drought stress compared to genes in LK3 (the drought-sensitive genotype of *Tartary* buckwheat) (Meng et al., 2022). WGCNA analysis identified 22 distinct co-expression modules, among which the deep blue module exhibited the strongest positive association with drought tolerance characteristics. Further analysis identified twenty potential hub genes, including g47370 (AFP2), g14296 (CDKF), and g60091 (SPBC2A9), which were found to be potentially associated with drought resistance regulation in sweet potato (Zong et al., 2023). WGCNA revealed *MsC3H29c* as a key drought-responsive gene: its overexpression increased primary root length and biomass in Medicago sativa, whereas RNAi silencing reduced these traits (Dong et al., 2024).

With ongoing advances in cotton molecular biology and functional genomics, researchers have uncovered key molecular pathways involved in drought response and identified several drought-tolerant genes. Nevertheless, the comparative analysis of adaptive mechanisms among different drought-resistant cotton genotypes remains to be fully elucidated. For example: (1) Systematic comparison of gene expression and metabolite dynamics under drought stress in different drought-resistant varieties is insufficient; (2) The association network between variety-specific differential genes and metabolites is not clear; (3) The key regulatory modules and hub genes that confer high drought resistance to varieties have not been fully mined. At the same time, traditional WGCNA mostly relies on morphological or physiological phenotypes (such as plant height, MDA, proline content, SOD activity, etc.) for module selection (Tang et al., 2023; Wang et al., 2024a). There is a lack of research on the correlation analysis between metabolomic data and gene expression modules.

In this study, upland cotton variety 64-22–3 with strong drought resistance and drought-sensitive variety A3 were used as materials. Integrated transcriptomic and metabolomic analysis was used to systematically compare the DEGs and DAMs of 64-22–3 and A3 in the control and drought groups. KEGG analysis showed that Phenylpropanoid biosynthesis, Linoleic acid metabolism, and Glucosinolate biosynthesis were specifically and significantly enriched in the drought-resistant variety. We further conducted WGCNA analysis on drought-specific DEGs and DAMs in the drought-resistant cultivar 64-22–3 under water deficit conditions. This identified two co-expression modules that exhibited strong positive correlations with the unique accumulation of drought-responsive metabolites in cultivar 64-22-3, with genes *Ghi_D06G05631* and *Ghi_A13G12271* serving as hub genes for these respective modules, which encodes TOPLESS-related 1 protein and CIPK6 (CBL-interacting protein kinase 6) separately. These genes may be key factors that confer stronger stress adaptation capabilities to drought-resistant varieties. The study advances our understanding of drought resistance mechanisms in cotton and reveals candidate genes for breeding enhanced tolerance.

## 2 Materials and methods

### 2.1 Experimental materials

Plant materials included ten cotton accessions provided by the National Medium-term Gene Bank of Cotton in China and the National Cotton Germplasm Resources Platform. This included drought-sensitive varieties such as Fandimian (Fd), Zhong 6429 (Z6429), Anmian 1 (A1), Anmian 3 (A3), and Wanmian 3 (W3); as well as drought-resistant materials including Zhong 833 (Z833), Dongfeng 4 (D4), Xinmian 291 (X291), Changde 184 (C184), and 64-22-3.

Initially, cotton seeds were subjected to sulfuric acid defuzzing treatment, followed by drying. Seeds that were plump and had a darker color were selected and soaked in 15% sodium hypochlorite-based solution for 15 minutes, then thoroughly washed with sterile water. Subsequently, the seeds were soaked in the dark at 25°C for 12 hours and then transferred to a germination environment with a temperature of 25°C and a 12-hour photoperiod for 24–36 hours. Seeds with consistent radicle lengths were chosen for planting. At the one-leaf-one-heart stage, uniform healthy cotton seedlings were chosen, secured with sponges, and transplanted to pots filled with half-strength Hoagland solution for hydroponic growth. The nutrient solution was renewed with fresh half-strength Hoagland solution every 3–4 days.

### 2.2 Drought treatment

For hydroponic cultivation, 10%, 15%, and 20% PEG6000 solutions were initially prepared to pre-treat the cotton materials. Based on the observed phenotypic responses of the cotton plants, a 15% PEG6000 solution was selected to simulate the drought stress environment. When the cotton seedlings reached the two-leaf-one-heart stage, 15% PEG6000 solution was added to the culture medium to initiate the stress treatment. The wilting degree and survival status of the cotton plants were observed and photographed at 0h, 3h, 6h, 12h, 24h, and 36h post-treatment.

For soil cultivation, 3-week-old cotton plants with consistent growth status were selected for both the control and drought groups. Before drought treatment, pots with equal amounts of soil were watered quantitatively. Subsequently, natural drought treatment was applied. Once distinct phenotypic differences emerged between the control and drought groups, immediate photographic records were taken.

### 2.3 Measurement of indicators

Cotton plants were subjected to simulated drought stress using a 15% PEG6000 solution. The wilting degree and survival status of the plants were monitored at 0h, 3h, 6h, 12h, 24h, and 36h post-treatment. After the 36h treatment, the relative water content, maximum photochemical efficiency of photosystem II (Fv/Fm), and root-shoot ratio of the cotton plants were measured. Three cotton seedlings were used as one biological replicate for these measurements.

The degree of drought stress on cotton plants was assessed using the absolute soil water content (ASWC), calculated as follows: ASWC (%) = [(Pre-drought total weight of the soil-pot system) - (dry weight of mixed nutrient soil + pot weight))/dry weight of mixed nutrient soil] × 100%. When the ASWC reached approximately 10%, the plants were considered to be under moderate drought stress. Once the cotton plants reached this moderate drought level, samples were collected to measure physiological indicators such as peroxidase (POD), superoxide dismutase (SOD), catalase (CAT), malondialdehyde (MDA), and proline (Pro). These measurements were conducted using reagent kits from Suzhou Greiss Biotechnology Co., Ltd., following the operational procedures outlined in the kit instructions.

### 2.4 Experimental workflow (RNA-seq analysis)

Total RNA isolated from cotton leaves was reverse transcribed for cDNA library preparation. The libraries were sequenced using Illumina HiSeq2000, generating raw sequencing data that underwent quality control to yield high-quality reads. These reads were mapped to the *Gossypium hirsutum* reference genome (TM-1_WHU) with TopHat2, followed by transcript assembly using StringTie. Functional annotation was performed through BLAST searches against NR, Swiss-Prot, GO, COG, KOG, and KEGG databases. Gene expression levels were quantified with RSEM, and differential expression analysis was performed using DESeq2 (R package) with thresholds of fold change (FC) ≥ 2 and False Discovery Rate (FDR) < 0.05. DEGs were mapped to GO terms and KEGG pathways, followed by hypergeometric tests to determine significantly enriched functional categories and metabolic/signaling pathways relative to the genomic background.

### 2.5 Metabolomic analysis

Leaf samples (100 mg) were cryogenically ground in liquid nitrogen and extracted with 500 μL 80% methanol/water (v/v). Following vortexing and 5 min incubation on ice, centrifugation was performed at 15,000g (4°C, 20 min). The supernatant was diluted to 53% methanol with LC-MS grade water, centrifuged again under the same conditions, and analyzed by LC-MS. Raw data were converted to mzXML format (ProteoWizard) and processed using XCMS (10 ppm mass tolerance) for feature detection, retention time alignment, and area normalization. Metabolite identification was achieved by matching MS2 spectra against reference databases (e.g., HMDB, MassBank), with background subtraction using blank samples. Data normalization was performed prior to statistical analysis, and all computational workflows were implemented on CentOS 6.6 using R/Python scripts. DAMs were identified through integrated multivariate (OPLS-DA) and univariate (Student’s t-test) analyses. Metabolites meeting dual criteria (VIP ≥1 from OPLS-DA and *p*<0.05 from t-tests) were considered significant. These DAMs were then annotated against the KEGG database, with pathway enrichment assessed via hypergeometric testing relative to the complete metabolic background.

### 2.6 Integrated transcriptomic and metabolomic analysis using WGCNA

Weighted Gene Co-expression Network Analysis (WGCNA) was employed to systematically integrate transcriptomic and metabolomic data. Co-expression modules of genes were identified, and key modules were selected based on their correlation strengths with phenotypic traits. Hub genes and key transcription factors (TFs) within these modules were identified based on intramodular connectivity for subsequent analysis. A gene interaction network was constructed using Cytoscape software to visualize the co-expression relationships between hub genes, key TFs, and other correlated genes.

## 3 Results

### 3.1 Drought-resistant variety screening

In this study, a 15% PEG6000 solution was utilized to simulate drought stress conditions for treating cotton seedlings. Phenotypic observations revealed that after 36 hours of stress treatment, four varieties—Fd (Fandi Cotton), A1 (Anmian 1), A3 (Anmian 3), and W3 (Wanmian 3)—exhibited significant wilting and dehydration. In comparison, three lines—Z833 (Zhong 833), C184 (Changde 184), and 64-22-3 —maintained relatively good growth conditions (**Supplementary Figure 1**). Based on these phenotypic differences, seven typical materials were ultimately selected for further analysis: four drought-sensitive varieties (sensitive group) and three drought-resistant varieties (resistant group). The sensitive group included Fd, A1, A3, and W3, while the resistant group comprised Z833, C184, and 64-22-3.

**Figure 1:**
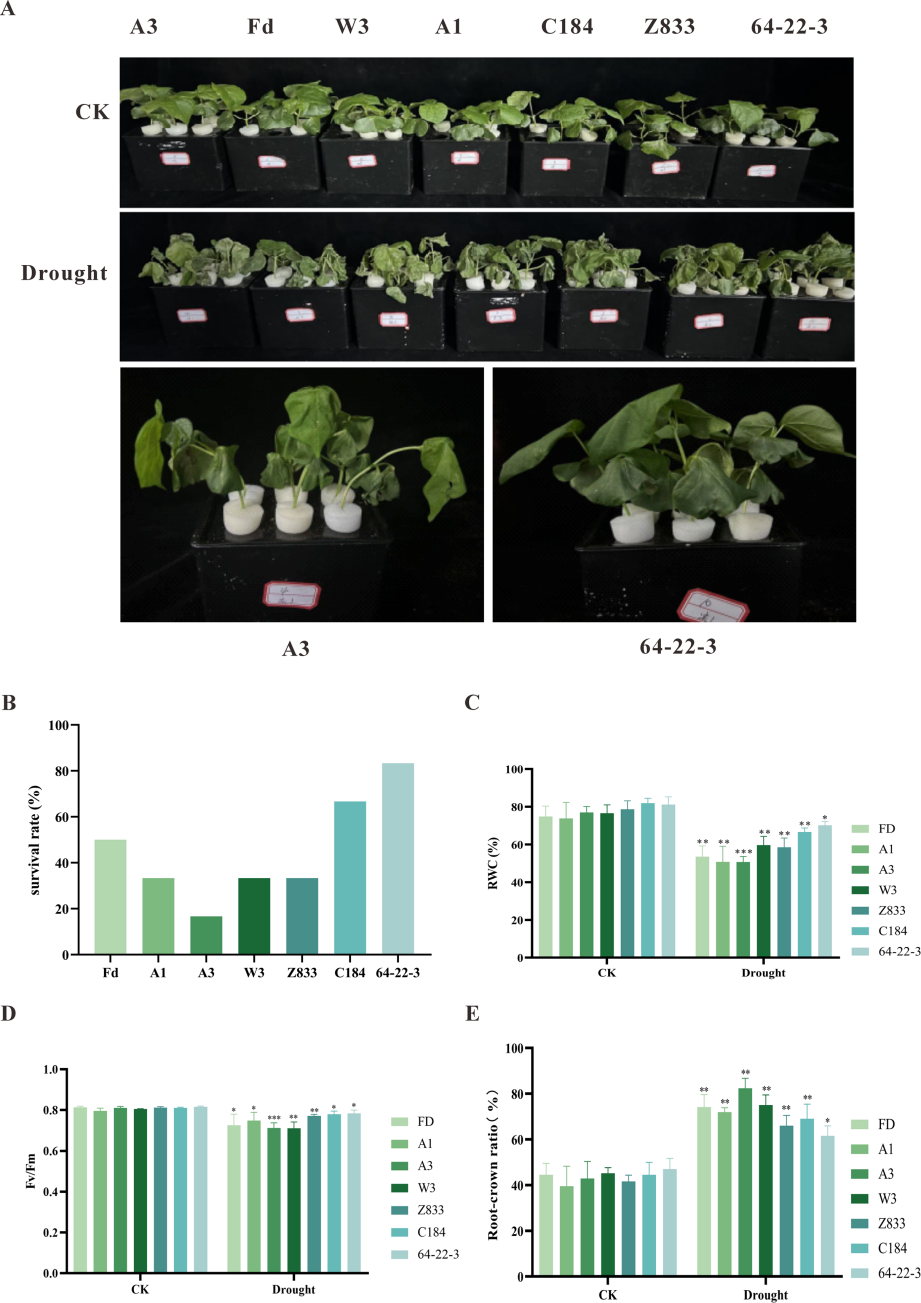
Phenotypic and physiological characterization of different cotton cultivars under normal irrigation (CK) and drought stress conditions(Drought). Asterisks (*) indicate significant differences (*p* < 0.05) between drought-treated and control groups within each cultivar. **(A)** Phenotypic responses of cotton cultivars to PEG6000-simulated drought stress; **(B)** Survival rates of different cotton cultivars under drought stress; **(C)** Leaf relative water content (RWC) of cotton cultivars under normal irrigation and drought treatment; **(D)** Maximum quantum yield of PSII photochemistry (Fv/Fm) in cotton cultivars under both conditions; **(E)** Root-to-shoot ratio of cotton cultivars under normal irrigation and drought stress.

To further validate the results of the initial experiment and to identify the most drought-resistant and drought-sensitive varieties, a re-screening experiment was conducted using a 15% PEG6000 solution to simulate drought stress. The results indicated that under normal watering conditions, there were no significant differences in growth indices and physiological characteristics among the different lines. However, after drought stress treatment, significant differences in stress resistance were observed among the varieties (**Figure 1A**). Specifically, A3 exhibited severe wilting and even plant death after 36 hours of stress, resulting in a survival rate of only 16.3%. In comparison 64-22–3 demonstrated strong drought resistance, with a survival rate of 80% and relatively intact plant morphology (**Figure 1B**). Physiological assessments confirmed varietal differences in drought tolerance. While all genotypes showed reduced leaf RWC under water deficit, the drought-resistant 64-22–3 exhibited a smaller decline (11%) compared to the sensitive A3 (26%) (**Figure 1C**). Similarly, chlorophyll fluorescence analysis revealed that 64-22–3 maintained higher Fv/Fm values (3.2% reduction) than A3 (9.9% decrease) following drought stress (**Figure 1D**). Additionally, analysis of the root-to-shoot ratio indicated that drought stress enhanced root development in all lines, with 64-22–3 showing a significantly greater increase in root-to-shoot ratio (39%) compared to A3 (11%). This suggests that 64-22–3 has enhanced water acquisition ability through root system restructuring (**Figure 1E**). Based on a comprehensive analysis of phenotypic and physiological indices, A3 was ultimately determined to be a typical drought-sensitive variety, while 64-22–3 was identified as a highly drought-resistant variety. These two varieties, representing an extreme contrast in stress resistance phenotypes, provide ideal material for subsequent studies on drought resistance mechanisms.

**Figure 2:**
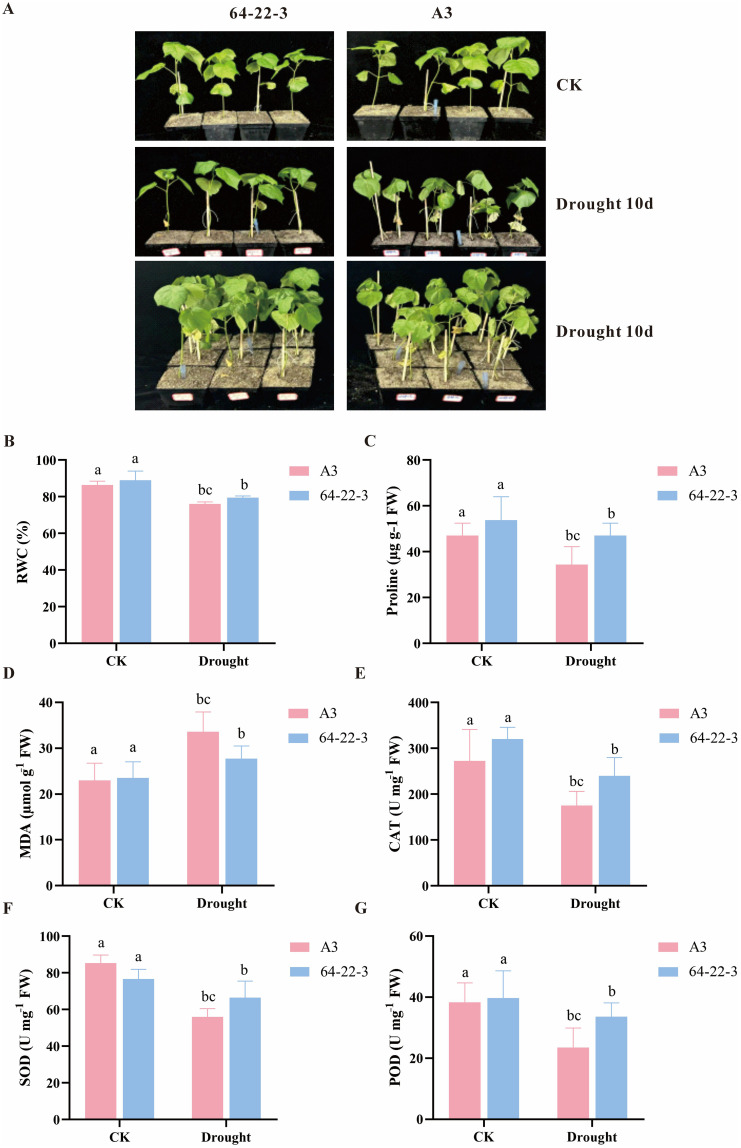
Phenotypic and physiological characterization of cotton under normal irrigation and drought conditions. Different lowercase letters indicate significant differences among treatment groups at *p* < 0.05 level. **(A)** Phenotypic comparison between normally irrigated and drought-stressed cotton plants; **(B)** Leaf relative water content (RWC, %) in control and drought-treated groups; **(C)** Proline content (Pro) under normal irrigation and drought stress; **(D)** Malondialdehyde content (MDA) in control and drought-exposed plants; **(E)** Catalase (CAT) activity in cotton under well-watered and drought-stress conditions. **(F)** Superoxide dismutase (SOD) content in cotton under well-watered and drought-stress conditions. **(G)** Peroxidase (POD) activity in cotton under well-watered and drought stress conditions.

Following screening of materials with contrasting phenotypes, the drought-sensitive A3 and drought-tolerant 64-22–3 varieties were selected for soil-based drought experiments. At the two-leaf-one-heart stage, plants were subjected to water deficit while controls received normal irrigation. After 10 days of treatment, A3 displayed severe leaf wilting compared to the mild symptoms observed in 64-22-3 (**Figure 2A**). Post-drought analysis revealed significantly greater leaf RWC in 64-22–3 compared to A3 (*p*<0.05), confirming its superior drought tolerance (**Figure 2B**). The accumulation of proline (Pro) in the leaves of 64-22–3 was significantly higher than that in A3, suggesting a stronger osmotic regulation ability (**Figure 2C**). Moreover, the malondialdehyde (MDA) content in A3 was significantly higher than that in 64-22-3, indicating more severe lipid peroxidation damage to the cell membranes (**Figure 2D**). Analysis of the antioxidant enzyme system revealed that the activities of catalase (CAT), superoxide dismutase (SOD), and peroxidase (POD) in 64-22–3 were all significantly higher than those in A3, confirming that 64-22–3 maintained cellular homeostasis by enhancing its ability to scavenge reactive oxygen species (**Figures 2E-G**). Furthermore, transcriptomic analysis indicated that under drought conditions, the drought-resistant cultivar 64-22–3 exhibited significantly upregulated expression of multiple antioxidant enzyme-related genes, including Peroxidase(PRX), peroxidase(POD), Superoxide Dismutase(SOD), Ascorbate Peroxidase(APX), compared to cultivar A3 (**Supplementary Figure 2**). This suggests a more robust antioxidant response mechanism, which may be closely associated with enhanced drought resistance in cotton. Collectively, these results suggest that cultivar 64-22–3 enhances drought tolerance through synergistic strategies involving osmoprotectant accumulation, alleviation of membrane damage, and strengthening of antioxidant enzyme activity.

### 3.2 Transcriptomic sequencing analysis

To further investigate the molecular mechanisms underlying the drought stress response in cotton varieties 64-22–3 and A3, we conducted transcriptomic sequencing on the leaves of these plants under both normal irrigation and drought treatment conditions. Quality analysis of the sequencing data revealed Q30 values ranging from 93.54% to 95.09%, with GC content between 42.77% and 44.33%. (**Supplementary Table S1**). These results demonstrate that we obtained high-quality transcriptomic data suitable for further analysis.

### 3.3 Identification and analysis of differentially expressed genes

**Figure 3:**
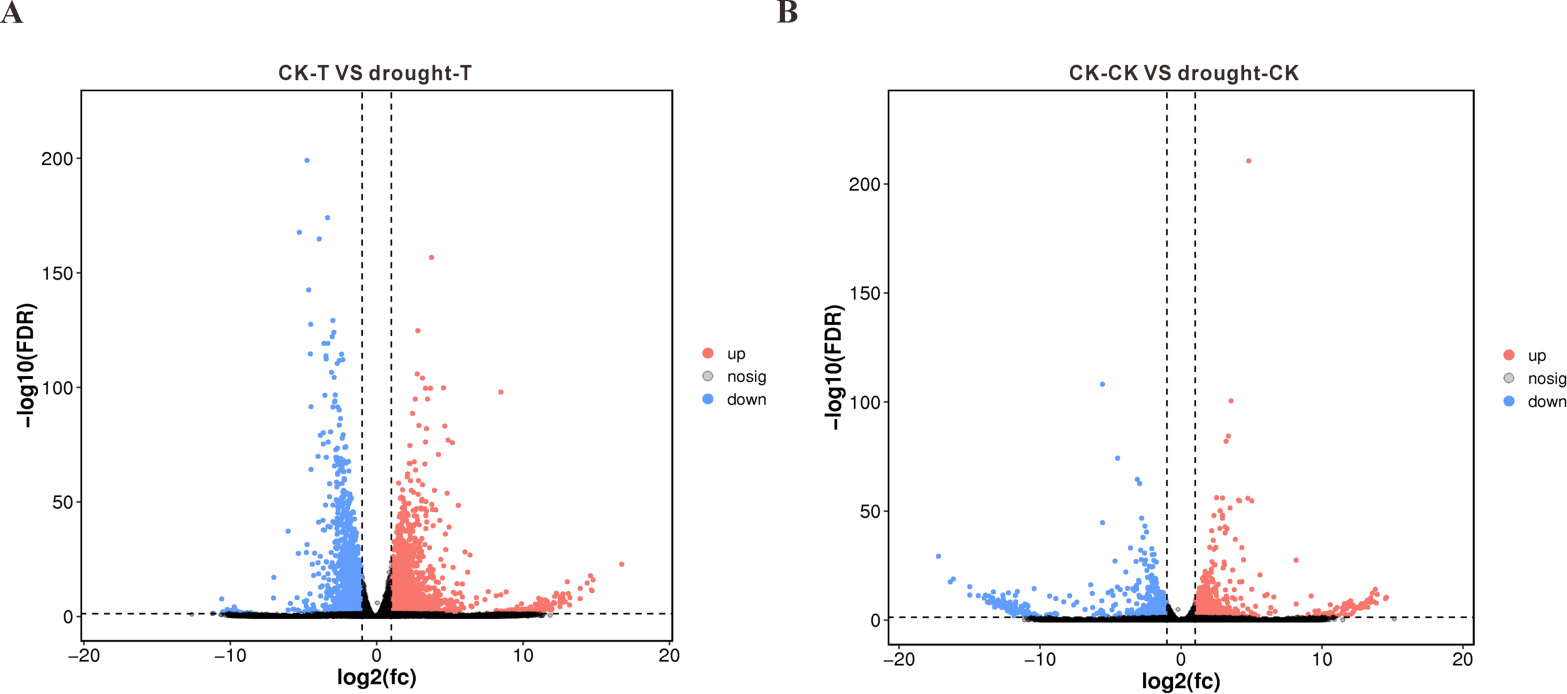
Volcano plots of differentially expressed genes (DEGs) between drought-stressed and well-watered conditions in drought-tolerant cultivar 64-22–3 and drought-sensitive cultivar A3. The x-axis represents log2(FC) of gene expression between two groups, while the y-axis shows -log10(FDR) values. Red dots indicate significantly upregulated genes (Drought vs CK, FDR < 0.05 and log2(FC) ≥ 1), blue dots represent significantly downregulated genes (FDR < 0.05 and log2(FC) ≤ -1), and black dots denote non-significant genes. **(A)** DEG volcano plot for drought-tolerant cultivar 64-22-3; **(B)** DEG volcano plot for drought-sensitive cultivar A3.

To elucidate transcriptional responses to drought across varieties with differential resistance, we identified DEGs (*p*<0.05, |log2FC|>1). Comparative analysis revealed 7,351 significantly altered genes in the drought-tolerant 64-22–3 cultivar under water deficit versus well-watered conditions, with 5,034 genes upregulated and 2,317 genes downregulated (**Figure 3A**). In comparison, the drought-sensitive variety A3 showed significant changes in the expression of 5,009 genes under drought stress, with 3,222 genes upregulated and 1,787 genes downregulated (**Figure 3B**). We found that the drought-resistant variety 64-22–3 had a significantly higher number of DEGs compared to the drought-sensitive variety A3 under drought treatment conditions. This suggests that the drought-resistant variety 64-22–3 is capable of mobilizing a greater number of genes to participate in the drought stress response. Additionally, we observed that a higher proportion of DEGs in the drought-resistant variety were upregulated, accounting for 68% of the total DEGs, compared to 64% in the drought-sensitive variety. This may indicate that the drought-resistant variety enhances its drought tolerance by upregulating a larger number of genes.

### 3.4 Gene ontology enrichment analysis reveals divergent drought response mechanisms in cotton varieties

**Figure 4:**
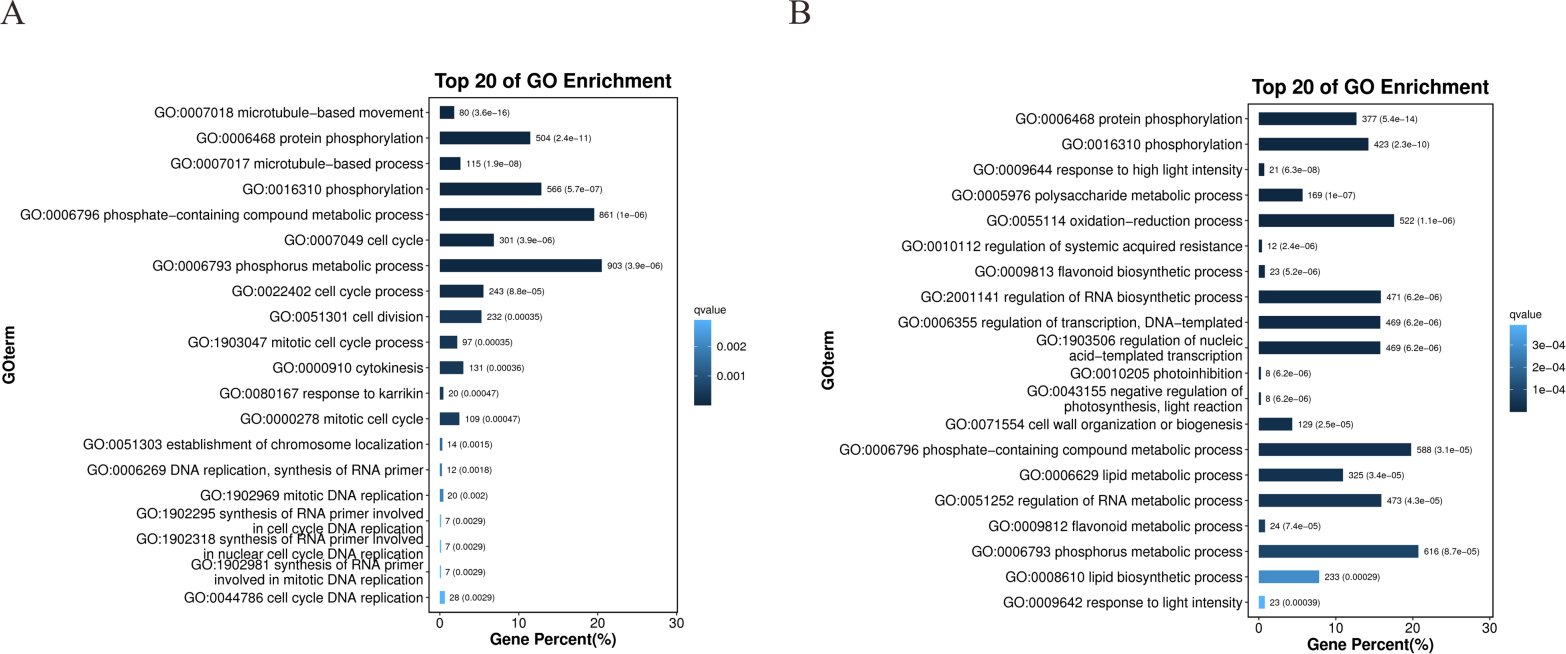
Gene Ontology (GO) enrichment analysis of DEGs between drought-stressed and well-watered conditions in drought-tolerant cultivar 64-22–3 and drought-sensitive cultivar A3. All enriched terms were selected with a threshold of *p*-value ≤ 0.05. **(A)** GO enrichment analysis of biological processes in drought-tolerant cultivar 64-22-3; **(B)** GO enrichment analysis of biological processes in drought-sensitive cultivar A3.

GO enrichment analysis of DEGs highlighted distinct functional patterns between drought-resistant (64-22-3) and drought-sensitive (A3) cotton varieties. Cellular component analysis revealed significant enrichment of DEGs in chromosomal and cytoskeletal organization pathways within the drought-resistant line (e.g., FANCM-MHF complex, nucleosome, mitotic spindle midzone), whereas sensitive-line DEGs enriched membrane-related terms (e.g., apoplast, chloroplast envelope) (**Supplementary Figures 3A, B**). For molecular function, resistant-line DEGs showed strong involvement in microtubule binding and ATP-dependent kinase activities (e.g., protein kinase, phosphotransferase), while sensitive-line DEGs were linked to oxidoreductase and glycosyl/phosphoryl transferase activities (**Supplementary Figures 3C, D**). Biological process analysis revealed that resistant-line DEGs primarily participated in microtubule-based movement, cell cycle regulation, and DNA replication, suggesting active cellular restructuring under stress (**Figure 4A**). In comparison, sensitive-line DEGs were enriched in polysaccharide metabolism, redox processes, and transcriptional regulation. Strikingly, both varieties shared enrichment in phosphorylation-related processes, underscoring their conserved role in drought response (**Figure 4B**). The resistant variety’s unique engagement of cytoskeletal and cell-cycle pathways (e.g., microtubule motor activity, chromosome localization) implies a potential mechanistic basis for enhanced drought tolerance, aligning with established roles of cytoskeletal dynamics in stress adaptation.

### 3.5 Metabolomic sequencing of 64-22-3 and A3 under drought stress

To compare drought-responsive metabolic networks between cotton cultivars 64-22–3 and A3, we performed LC-MS/MS-based untargeted metabolomics on leaf samples under water deficit conditions. Quality assessment revealed excellent chromatographic stability in the total ion current (TIC) profiles of QC samples, while principal component analysis (PCA) showed that biological replicates clustered closely within each group, with clear separation between distinct sample groups (**Supplementary Figure 4**). A total of 1,500 metabolites were identified across all samples. These metabolites included: Alkaloids and derivatives (37), Benzenoids (164), Hydrocarbon derivatives (3), Hydrocarbons (6), Lignans, neolignans, and related compounds (19), Lipids and lipid-like molecules (454), Nucleosides, nucleotides, and analogues (13), Organic acids and derivatives (183), Organic nitrogen compounds (21), Organic oxygen compounds (94), Organohalogen compounds (1), Organoheterocyclic compounds (286), and Phenylpropanoids and polyketides (202), Acetylides(1),Organohalogen compounds(1),Organosufur compounds(5).

### 3.6 Identification of differential metabolites

**Figure 5:**
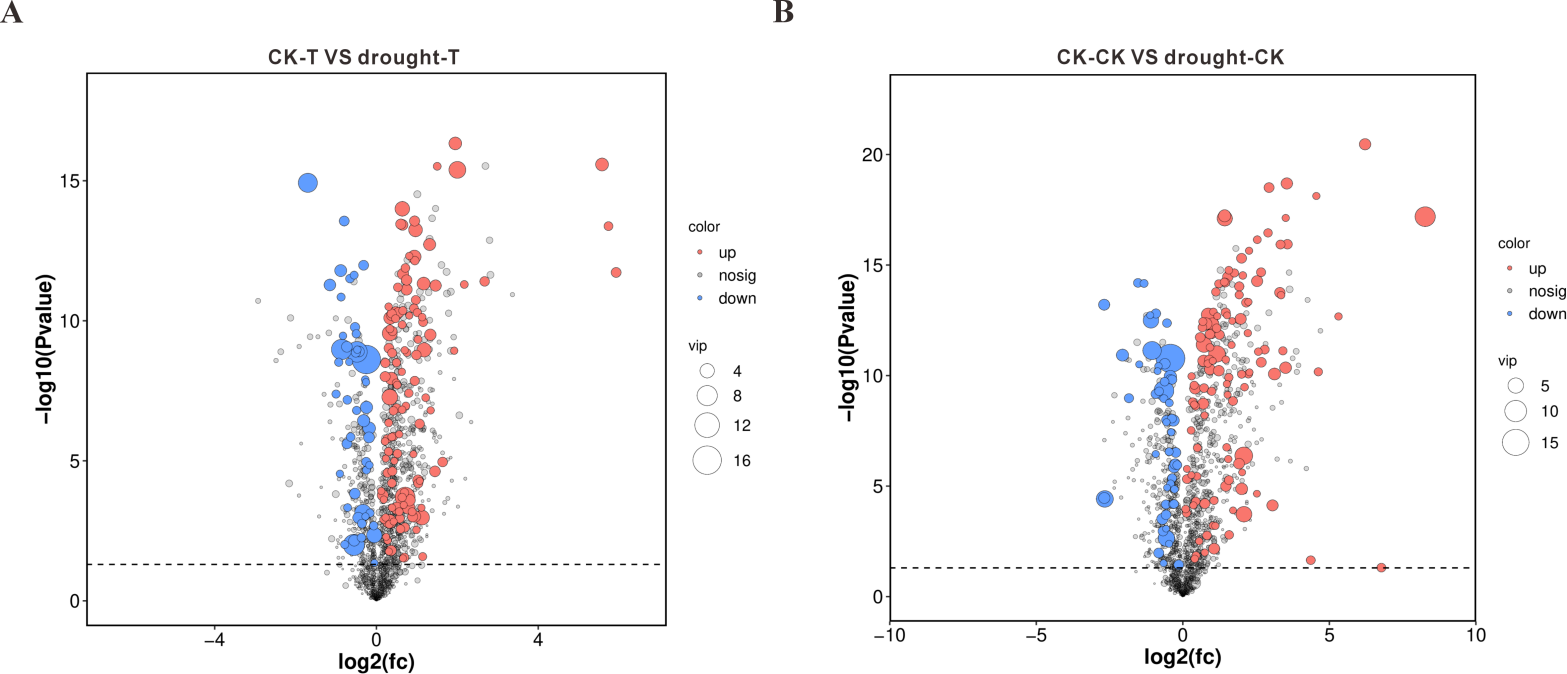
Volcano plots of differential metabolites between drought-stressed and well-watered conditions in drought-tolerant cultivar 64-22–3 and drought-sensitive cultivar A3. The x-axis represents log2(FC) of metabolite abundance between comparison groups, while the y-axis shows -log10-transformed *p*-values. The dashed horizontal line indicates the *p*-value threshold for screening differential metabolites. Red dots denote significantly upregulated metabolites (VIP ≥ 1 and *p* < 0.05 with FC > 1), blue dots represent significantly downregulated metabolites (VIP ≥ 1 and P < 0.05 with FC < -1), and dot size corresponds to VIP value magnitude. **(A)** Differential metabolite volcano plot for drought-tolerant cultivar 64-22-3; **(B)** Differential metabolite volcano plot for drought-sensitive cultivar A3.

To explore the changes in metabolites during drought stress, we established criteria for differentially accumulated metabolites (DAMs) as VIP > 1 and *P*-value < 0.05. Under drought stress conditions, we identified 169 DAMs in the 64-22–3 variety (120 upregulated and 49 downregulated) (**Figure 5A**), while 173 DAMs were identified in the A3 variety (120 upregulated and 53 downregulated) (**Figure 5B**).

### 3.7 KEGG co-enrichment analysis unveils metabolome-transcriptome linkage patterns

**Figure 6:**
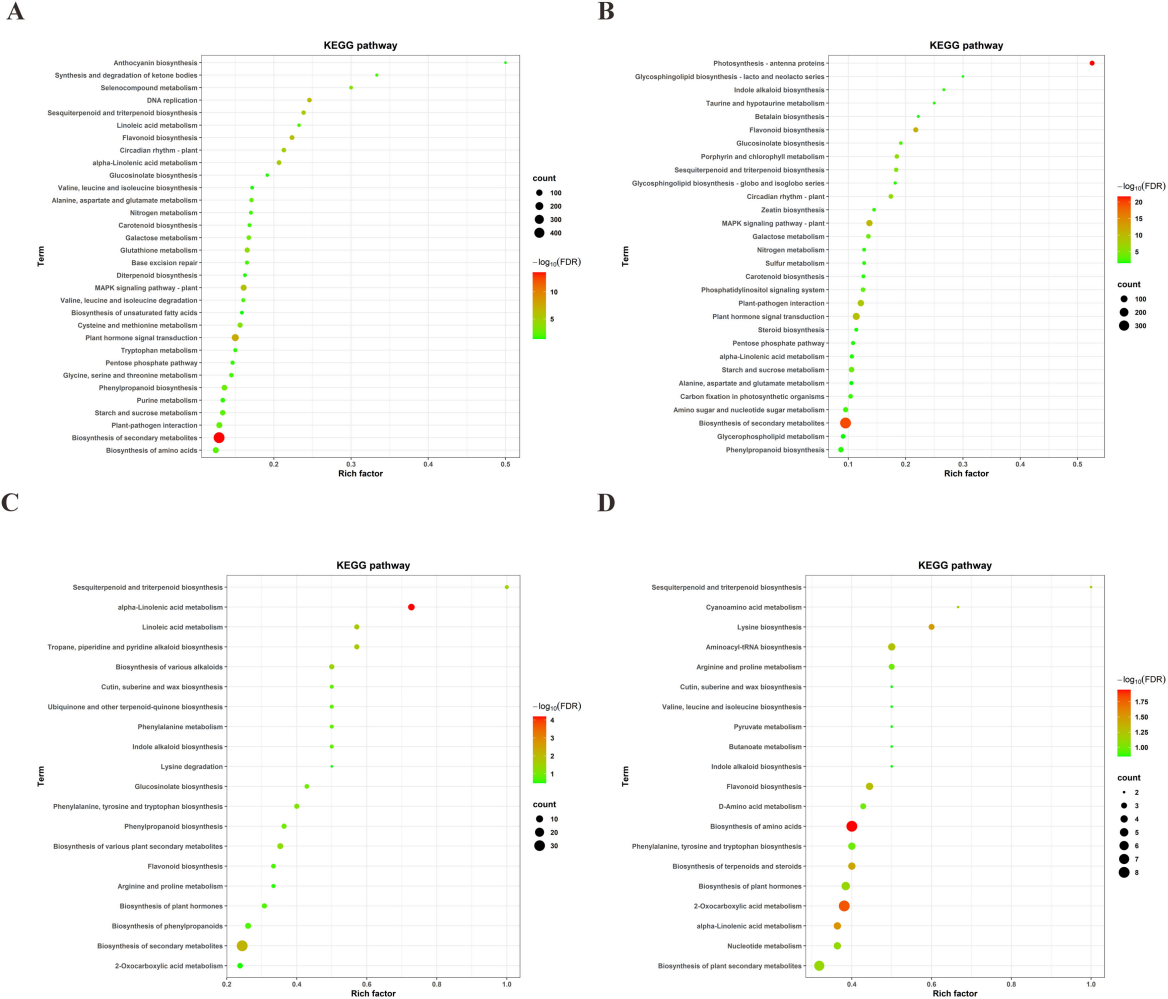
KEGG enrichment analysis of DEGs and DAMs between drought-stressed and well-watered conditions in drought-tolerant cultivar 64-22–3 and drought-sensitive cultivar A3. **(A)** KEGG pathway enrichment of DEGs in drought-tolerant cultivar 64-22-3; **(B)** KEGG pathway enrichment of DEGs in drought-sensitive cultivar A3; **(C)** KEGG pathway enrichment of DAMs in drought-tolerant cultivar 64-22-3; **(D)** KEGG pathway enrichment of DAMs in drought-sensitive cultivar A3.

Pathway enrichment analysis of the DEGs revealed that in the 64-22–3 variety, the significantly enriched pathways included biosynthesis of secondary metabolites, plant hormone signal transduction, DNA replication, flavonoid biosynthesis, MAPK signaling pathway, alpha-linolenic acid metabolism, circadian rhythm in plants, sesquiterpenoid and triterpenoid biosynthesis, glutathione metabolism, selenocompound metabolism, cysteine and methionine metabolism, galactose metabolism, phenylpropanoid biosynthesis, alanine, aspartate, and glutamate metabolism, plant-pathogen interaction, starch and sucrose metabolism, biosynthesis of amino acids, and linoleic acid metabolism (**Figure 6A**). In comparison, the DEGs of the A3 variety were mainly enriched in the following pathways: photosynthesis - antenna proteins, biosynthesis of secondary metabolites, flavonoid biosynthesis, MAPK signaling pathway in plants, plant hormone signal transduction, plant-pathogen interaction, circadian rhythm in plants, porphyrin and chlorophyll metabolism, sesquiterpenoid and triterpenoid biosynthesis, starch and sucrose metabolism, galactose metabolism, phosphatidylinositol signaling system, glucosinolate biosynthesis, amino sugar and nucleotide sugar metabolism, and carbon fixation in photosynthetic organisms (**Figure 6B**). The KEGG pathways that were significantly enriched in the DEGs of both the drought-resistant and drought-sensitive varieties included the biosynthesis of secondary metabolites, flavonoid biosynthesis, plant hormone signal transduction, MAPK signaling pathway, plant-pathogen interaction, circadian rhythm in plants, sesquiterpenoid and triterpenoid biosynthesis, starch and sucrose metabolism, and galactose metabolism. These metabolic pathways represent evolutionarily conserved components of cotton’s drought response. Notably, the tolerant variety 64-22–3 showed distinct DEG enrichment in multiple pathways including: α-linolenic acid metabolism, glutathione cycling, selenocompound processing, sulfur amino acid metabolism, phenylpropanoid biosynthesis, nitrogenous amino acid metabolism, amino acid biosynthesis, and linoleic acid metabolism, potentially contributing to its enhanced drought adaptation.

KEGG pathway enrichment analysis was performed on the DAMs identified within the drought-resistant cultivar 64-22–3 and within the drought-sensitive cultivar A3, respectively. Under drought stress, the DAMs that accumulated in the drought-resistant variety 64-22–3 were significantly enriched in the following pathways: biosynthesis of phenylpropanoids, aminoacyl-tRNA biosynthesis, nucleotide metabolism, arginine and proline metabolism, flavonoid biosynthesis, phenylalanine metabolism, 2-oxocarboxylic acid metabolism, sesquiterpenoid and triterpenoid biosynthesis, D-amino acid metabolism, linoleic acid metabolism, C5-branched dibasic acid metabolism, biosynthesis of secondary metabolites, and the biosynthesis of phenylalanine, tyrosine, and tryptophan, as well as lysine biosynthesis (**Figure 6C**). In comparison, the DAMs in the A3 variety showed significant enrichment in pathways such as amino acid biosynthesis, sesquiterpenoid and triterpenoid biosynthesis, aminoacyl-tRNA biosynthesis, flavonoid biosynthesis, plant hormone biosynthesis, plant secondary metabolite biosynthesis, phenylalanine/tyrosine/tryptophan biosynthesis, and terpenoid/steroid biosynthesis, cyanoamino acid metabolism, D-amino acid metabolism, alpha-linolenic acid metabolism, nucleotide metabolism, cutin, suberine, and wax biosynthesis, valine, leucine, and isoleucine biosynthesis, pyruvate metabolism, butanoate metabolism, and indole alkaloid biosynthesis (**Figure 6D**). We found that the KEGG pathways significantly enriched in the DAMs of both the drought-resistant and drought-sensitive varieties included 2-oxocarboxylic acid metabolism, sesquiterpenoid and triterpenoid biosynthesis, aminoacyl-tRNA biosynthesis, lysine biosynthesis, flavonoid biosynthesis, biosynthesis of plant secondary metabolites, arginine and proline metabolism, and phenylalanine, tyrosine, and tryptophan biosynthesis, along with D-amino acid metabolism and nucleotide metabolism (**Figures 6C, D**). These metabolic pathways represent conserved components of cotton’s drought response. The drought-tolerant cultivar 64-22–3 showed specific accumulation of DAMs in seven key pathways: 2-oxocarboxylic acid metabolism, sesquiterpenoid/triterpenoid biosynthesis, lysine production, flavonoid formation, arginine/proline cycling, aromatic amino acid biosynthesis, and nucleotide metabolism (**Figure 6C**).

Integrated transcriptomic and metabolomic analyses revealed significant co-enrichment in the resistant cultivar 64-22–3 for secondary metabolism, flavonoid biosynthesis, α-linolenic acid metabolism, sesquiterpenoid/triterpenoid biosynthesis, phenylpropanoid biosynthesis, linoleic acid metabolism, and glucosinolate biosynthesis (**Figures 6A, C**). In comparison, the DEGs and DAMs in the A3 variety showed significant enrichment in pathways including amino acid biosynthesis, sesquiterpenoid and triterpenoid biosynthesis, aminoacyl-tRNA biosynthesis, flavonoid biosynthesis, plant hormone biosynthesis, plant secondary metabolite biosynthesis, phenylalanine/tyrosine/tryptophan biosynthesis, and terpenoid/steroid biosynthesis (**Figures 6B, D**).

DEGs and DAMs in both cultivars were predominantly enriched in secondary metabolite biosynthesis, flavonoid biosynthesis, and sesquiterpenoid and triterpenoid biosynthesis (**Figures 6A–D**). Notably, the drought-resistant cultivar 64-22–3 exhibited specific DEGs and DAMs primarily involved in phenylpropanoid biosynthesis, linoleic acid metabolism, and glucosinolate biosynthesis. By constructing interaction networks between DEGs and DAMs within these pathways, we identified: 67 key genes (23 upregulated, 44 downregulated) and 4 metabolites (2 upregulated, 2 downregulated) in the phenylpropanoid biosynthesis pathway (**Supplementary Figure 5**); 10 key genes (all upregulated) and 4 metabolites (1 upregulated, 3 downregulated) in the linoleic acid metabolism pathway (**Supplementary Figure 6**); 9 key genes (6 upregulated, 3 downregulated) and 3 metabolites (all upregulated) in the glucosinolate biosynthesis pathway (**Supplementary Figure 7**). These findings provide novel insights into the molecular mechanisms underlying drought resistance in cotton and highlight potential target genes and metabolic markers for drought-resistant breeding.

### 3.8 Identification of key co-expression modules with hub genes and transcriptional regulators associated with drought resistance by WGCNA

**Figure 7:**
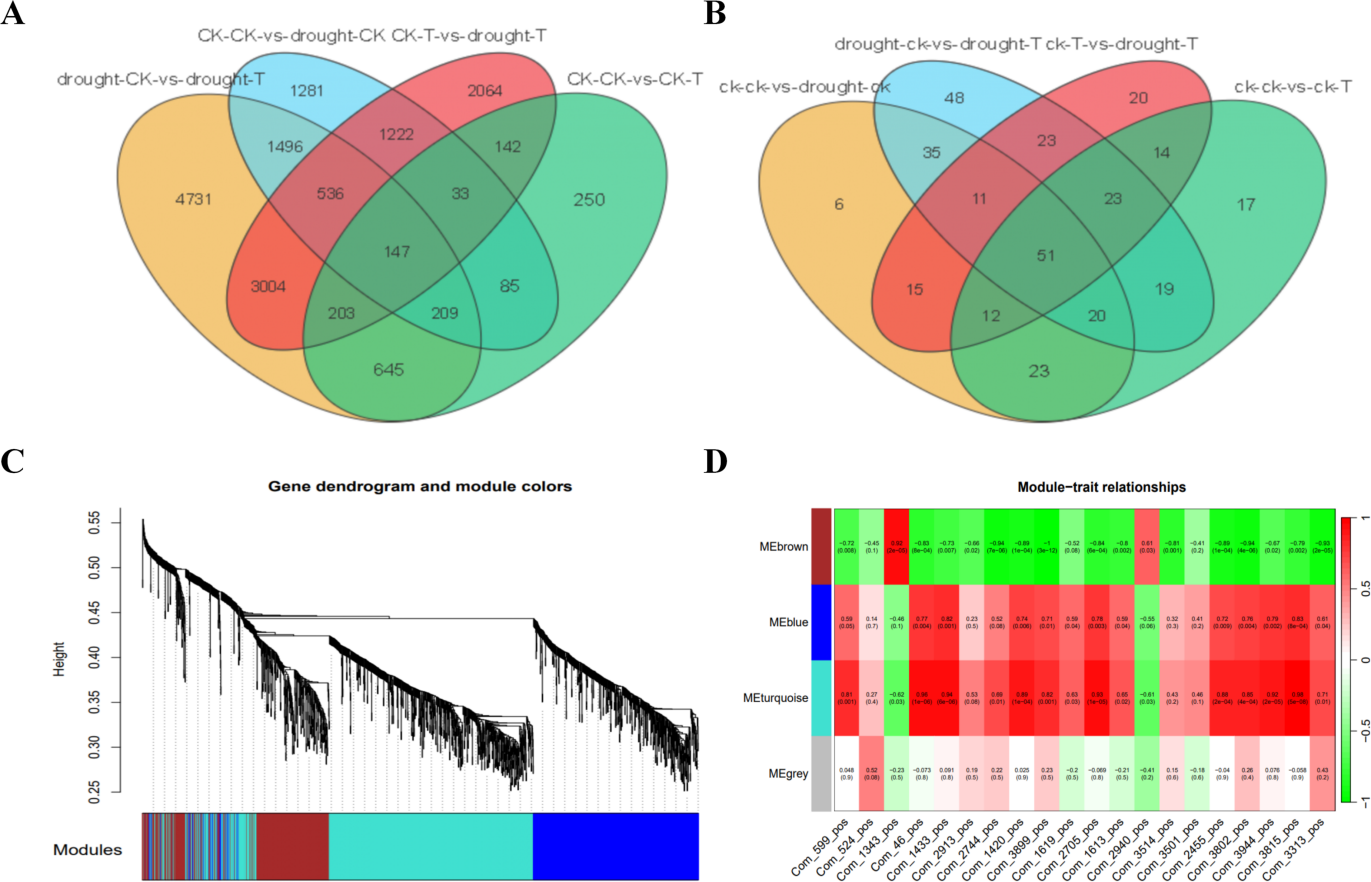
WGCNA of specific DAMs and DEGs in drought-resistant varieties under drought treatment conditions. **(A)** Venn diagram showing the number of shared and specific DEGs between different comparison groups; **(B)** Venn diagram showing the number of shared and specific DAMs between different comparison groups; **(C)** Hierarchical clustering dendrogram of genes with module color assignments; **(D)** Heatmap of correlations between different modules and various traits.

To further explore the molecular mechanisms underlying the enhanced drought resistance of the cotton variety 64-22-3, we performed differential expression and metabolite analysis between the two cotton varieties under both drought and normal irrigation conditions. A total of 1,714 DEGs, including 1,076 upregulated and 638 downregulated, were identified between the control groups of 64-22–3 and A3 (**Supplementary Figure 8A**). In addition, we found 10,971 DEGs (6,577 upregulated and 4,394 downregulated) between the drought-treated groups of 64-22–3 and A3 (**Supplementary Figure 8B**). Similarly, 173 DAMs (96 upregulated and 83 downregulated) were identified between the control groups (**Supplementary Figure 8C**), while 230 DAMs (123 upregulated and 107 downregulated) were detected between the drought-treated groups (**Supplementary Figure 8D**). Based on Venn diagram analysis, we screened out 2,064 genes and 20 metabolites that exhibited variety-specific differential expression and accumulation in 64-22–3 under drought stress compared to normal water conditions (**Figures 7A, B**). These findings suggest that these genes may regulate the accumulation of the 20 secondary metabolites, thereby contributing to the drought response of the drought-resistant variety and enhancing its drought resistance.

To systematically identify the gene regulatory networks that confer key drought resistance to the drought-resistant variety, we employed WGCNA in this study. A co-expression network was constructed based on the expression profiles of 2,064 variety-specific DEGs across 12 samples, and the quantitative data of 20 variety-specific DAMs were utilized as trait matrices for correlation analysis. The results indicated that two co-expression modules significantly associated with metabolites—the turquoise and blue modules—were identified (**Figure 7C**). Module eigengenes exhibited a strong correlation with metabolite accumulation levels (**Figure 7D**). Module-trait association analysis revealed that the blue and turquoise modules were closely related to the accumulation of the 20 metabolites, including benzenoids (4), lipids and lipid-like molecules (11), organic acids and derivatives (2), organic oxygen compounds (1), and phenylpropanoids and polyketides (2). These findings suggest that these modules may play important roles in the drought response of the drought-resistant variety.

**Figure 8:**
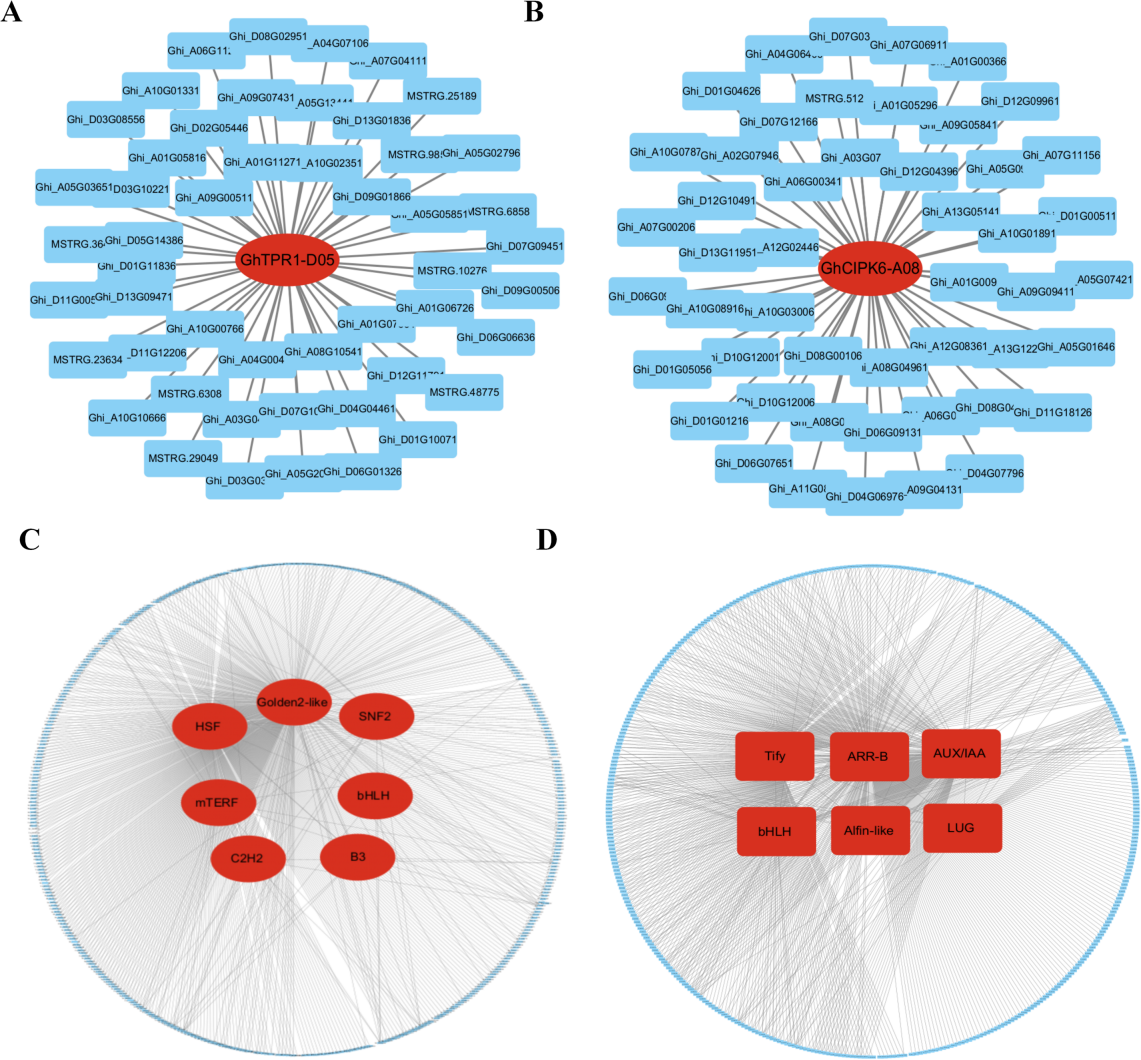
Identification of core genes in gene co-expression modules and screening of important transcription factors. **(A)** Hub gene *GhTPR1-D06* in the blue module, with lines representing the expression correlation between the hub gene and other genes. **(B)** Hub gene *GhCIPK6-A13* in the turquoise module, with lines representing the expression correlation between the hub gene and other genes. **(C)** Highly connected transcription factors (TFs) in the blue module, with lines indicating the expression correlation between transcription factors and other genes; **(D)** Highly connected core transcription factors (TFs) in the turquoise module, with lines indicating the expression correlation between transcription factors and other genes.

To further elucidate the key genes that confer enhanced drought resistance to the drought-resistant variety, we identified hub genes based on intramodular connectivity, module membership and gene significance. In the blue module, the core gene identified was *Ghi_D06G05631* (Module membership = 0.99), which encodes a TOPLESS-related 1 (TOPLESS) protein (**Figure 8A**). In the turquoise module, the hub gene was *Ghi_A13G12271* (Module membership = -0.99), annotated as CIPK6 (CBL-interacting protein kinase 6) (**Figure 8B**). qPCR validation of *Ghi_D06G05631* and *Ghi_A13G12271* revealed no significant differences in expression between well-watered and drought-treated samples in cultivar A3, whereas both genes exhibited significant expression changes under drought stress in the drought-tolerant line 64-22-3, which aligns with transcriptome profiling data (**Supplementary Figure 10**).

Further analysis revealed that the blue module contained seven classes of transcription factors with high connectivity, including HSF, Golden2-like, SNF2, mTERF, bHLH, C2H2, and B3 (**Figure 8C**). The turquoise module included six classes of highly connected transcription factors, mainly comprising Tify, ARR-B, AUX/IAA, bHLH, Alfin-like, and LUG (**Figure 8D**). These findings indicate that the blue and turquoise modules likely represent core regulatory networks maintaining drought resistance in cultivar 64-22-3. The genes *Ghi_D06G05631*, *Ghi_A13G12271*, and the aforementioned transcription factors are likely key players in conferring enhanced drought resistance to this variety.

## 4 Discussion

Drought stress impairs plant growth, triggering complex regulatory mechanisms for adaptation to arid conditions (He et al., 2024). Phenotypic and physiological assessments distinguished variety 64-22–3 as a drought-tolerant genotype and variety A3 as a drought-sensitive one, forming a suitable pair for comparative investigations. Further, a combined metabolomic and transcriptomic analysis strategy was employed to systematically elucidate the molecular regulatory networks underlying the response to drought stress in two cotton varieties (*Gossypium hirsutum L.*) with significant differences in drought tolerance and to reveal their variety-specific differences. WGCNA identified core gene modules exhibiting strong positive correlations with drought resistance, along with key hub genes and stress-responsive transcription factors. These results enhance our mechanistic understanding of cotton’s drought adaptation while offering novel candidate targets for breeding drought-tolerant cultivars.

Drought stress induces excessive reactive oxygen species (ROS) accumulation in plants, potentially causing oxidative cellular damage (Sato et al., 2024). To enhance their resistance to oxidative damage, plants have evolved a ROS scavenging system that includes enzymes such as SOD, POD, and CAT (Wang et al., 2025c). Under well-watered conditions, no significant differences in CAT, SOD, and POD activities were observed between cultivars 64-22–3 and A3. However, drought stress significantly elevated these enzymatic activities in the tolerant 64-22–3 compared to the sensitive A3. Transcriptomic analysis further demonstrated higher expression levels of multiple antioxidant genes (PRX, POD, SOD, APX) in drought-stressed 64-22–3 relative to stressed A3 (**Supplementary Figure 9**). These results demonstrate that the drought-tolerant 64-22–3 cultivar enhances ROS scavenging capacity through elevated antioxidant enzyme activity, mitigating oxidative cellular damage.

To further explore the differences in the molecular mechanisms underlying the response to drought stress between the drought-resistant variety 64-22–3 and the drought-sensitive variety A3, we conducted a combined transcriptomic and metabolomic analysis. Previous studies have demonstrated that the integration of transcriptomic and metabolomic approaches has become an effective means of elucidating the molecular mechanisms of plant responses to drought stress and identifying important drought-resistant genes (Zhu et al., 2024). The combined analysis revealed that *Agropyron mongolicum* primarily enhances its tolerance to drought stress through proline metabolism and the pentose phosphate pathway (Ma et al., 2024b). Integrated omics analysis demonstrated that drought stress induces resistance in soybean through *P5CS* and *PAO* gene upregulation, leading to increased proline and spermidine accumulation (Wang et al., 2024b). Drought-resistant goji (*Lycium barbarum*) enhances cuticular wax accumulation by upregulating key wax biosynthesis genes (e.g., *LbaWSD1* and *LbaCER1*) and transcription factors (e.g., *LbaMYB3)* under drought stress, leading to increased levels of wax components such as alcohols (C16), fatty acids (C19), and alkanes (C31 and C33). Consequently, it exhibits higher wax content and stronger drought tolerance compared to drought-sensitive varieties (Wang et al., 2024c). We found that both the DAMs and DEGs in the two varieties are enriched in the biosynthesis of secondary metabolites, flavonoid biosynthesis, and the biosynthesis pathways of sesquiterpenoids and triterpenoids (**Figures 6A–D**). Since flavonoids, sesquiterpenes, and triterpenes are all classified as secondary metabolites, this indicates that the biosynthesis pathways of secondary metabolites play a significant and conserved role in cotton’s response to drought stress (Baozhu et al., 2022). Existing research has demonstrated that PFG3 contributes significantly to drought adaptation and osmotic stress resistance through its regulatory function in flavonoid biosynthesis pathways. Drought stress triggers significant activation of flavonoid pathway genes in hybrid poplar, resulting in enhanced accumulation of phenolic and flavonoid compounds possessing antioxidant properties (Ahmed et al., 2021). Drought stress can also stimulate the biosynthesis pathways of terpenoid skeletons and triterpenoid compounds, thereby enhancing the synthesis of saikosaponins and improving plant drought resistance (Yang et al., 2021). Both our study and previous research have demonstrated that flavonoids, sesquiterpenes, and triterpenoids as secondary metabolites play a vital role in maintaining plant drought resistance.

Comparative analysis further demonstrated that drought-resistant cotton varieties show pronounced enrichment of DEGs and DAMs in phenylpropanoid biosynthesis, linoleic acid metabolism, and glucosinolate biosynthesis pathways (**Figure 6**). Specifically, exogenous application of tea polyphenols (a group of phenylpropanoid-derived compounds) significantly alleviated drought-induced damage in tea plants by activating the phenylpropanoid biosynthesis pathway (Sato et al., 2024). The *OsGRP3* gene in rice enhances drought resistance by regulating the phenylpropanoid biosynthesis pathway to promote lignin accumulation (Xu et al., 2022), which aligns with the pathway enrichment trends observed in this study. Drought stress can affect the fatty acid content in sunflowers (Ghaffari et al., 2023). Metabolomic analysis indicates that the linoleic acid metabolism pathway plays a role in the drought response of *Hibiscus mutabilis* (Zhang et al., 2024). Transcriptomic-proteomic data further demonstrated that 9-lipoxygenase, a key enzyme in the linoleic acid metabolism pathway, is upregulated at both transcriptional and translational levels under drought stress (Kim et al., 2024). Additionally, biohormones can improve broccoli’s drought tolerance by elevating glucosinolate levels (Montesinos et al., 2024). Allyl isothiocyanate (AITC), a hydrolysis product of glucosinolates, mitigates drought-induced growth inhibition in cabbage by modulating glucosinolate metabolism and inducing stomatal closure (Huang et al., 2024). Our findings further support the critical involvement of the phenylpropanoid biosynthesis, linoleic acid metabolism, and glucosinolate biosynthesis pathways in drought stress responses.

To further analyze the molecular mechanisms underlying the stronger drought resistance of drought-resistant variety 64-22-3, this study employed Venn diagram analysis and found that a total of 2064 genes and 20 metabolites exhibited significant differences in expression and accumulation between the drought treatment group and the control group in the drought-resistant variety 64-22-3. This suggests that these genes may enhance drought resistance by regulating the accumulation changes of these 20 metabolites. Notably, among these 20 metabolites, 9 showed more than a twofold increase in accumulation in the 64-22–3 line compared to the normal watering group, indicating their potential importance in the drought resistance mechanism. Analysis of key metabolites revealed that the accumulation of the isoflavonoid 2’-Hydroxygenistein 7-O-(6’’-malonylglucoside) was upregulated 44.67 times in the drought treatment group compared to the normal watering group. Under drought stress, it has been found that isoflavonoid accumulation can promote the reproductive growth phase of Iris to some extent (Ai et al., 2024). Additionally, a significant increase in isoflavonoid compounds in the roots was observed under drought conditions (Trush and Pal’ove-Balang, 2023). Our study found that isoflavonoid compounds significantly increased under drought stress, which is highly consistent with previous research results and further validates the important role of isoflavonoids in plant responses to drought. It was observed that the accumulation of the organooxygen compound Arnebinol was upregulated 4.7 times, and previous studies have shown that Arnebinol D can inhibit the production of aflatoxins in *Aspergillus flavus* (Madarshahi et al., 2022). Current studies report an association between Arnebinol and biotic stress. However, we found that its content was significantly upregulated under drought stress, suggesting its potential role in abiotic stress responses as well. Additionally, the accumulation of the steroid and steroid derivative 6-Desacetylscilliroside increased by 3.79 times, a finding that resonates with existing research. Under drought stress, it was demonstrated that treatment with 24-EBR (a bioactive steroid) enhanced both yield and oil content in safflower (Carthamus tinctorius) compared to the non-treated control (Zafari et al., 2020). Steroids in soybean play a critical role in mitigating oxidative stress and enhancing drought resistance, as evidenced in crops like soybean and wheat (Yu et al., 2024). These findings further highlight the pivotal function of steroid metabolites in mediating plant adaptation to drought.

WGCNA can identify gene modules and hub genes that are highly correlated with traits by constructing gene co-expression networks. It has become an effective tool for identifying important functional genes in plants. Researchers used WGCNA analysis to identify gene modules closely related to the relative water content of leaves, and screened out the module hub gene *ZmGRAS15* from them. Functional analysis showed that this gene positively regulates the drought response of maize (Wang et al., 2025a). WGCNA identified *PagNAC17* as a central regulator in poplar’s salt stress response. Transgenic overexpression of *PagNAC17* in 84K poplar substantially improved salt tolerance (Wang et al., 2025b). Heat stress (HS)-responsive transcriptome data identified three key genes, *PgCDF2*, *PgHSFA1*, and *PgHSFB3*, all of which play roles in the heat stress response of *Physalis grisea* (Jing et al., 2025). Most previous studies used growth index data or physiological index data as phenotypic data for WGCNA analysis (Feng et al., 2021; Li et al., 2022). This study conducted WGCNA using both quantitative data of 20 drought-specifically accumulated metabolites and 2,064 uniquely DEGs in the drought-resistant cultivar 64-22–3 under drought stress. The analysis revealed that two gene expression modules (Blue and Turquoise) exhibited a strong positive correlation with the accumulation of 20 metabolites (**Figure 7D**). Further screening of hub genes in these two modules identified *Ghi_D06G05631* as the hub gene in the blue module, which encodes the TOPLESS-related 1 protein (**Figure 8A**). It has been demonstrated that the interaction between *CmHSFA4*/*CmMYBS3* and *CmTPL* jointly inhibits the expression of *CmMYB121*, thereby enhancing salt stress tolerance in chrysanthemums (Wang et al., 2024d). Additionally, it has been found that *AtMYB44*, acting as a repressive transcription factor, regulates the expression of *APP2Cs*. The complex formed between *AtMYB44* and *TPR* represses PP2C transcription by promoting histone deacetylation at gene loci, thus participating in the transduction of the ABA signaling pathway (Nguyen and Cheong, 2018). Currently, there is no relevant literature reporting the direct role of the TOPLESS-related 1 protein in the drought stress response. Previous studies have established TOPLESS-related 1 protein’s involvement in plant salt stress responses and ABA signaling. Based on our findings, we hypothesize that TOPLESS-related 1 protein may similarly participate in drought stress adaptation. However, its precise functional role and regulatory mechanisms in cotton drought response remain to be elucidated. Notably, the turquoise module’s hub gene, *Ghi_A13G12271*, encodes CIPK6 (CBL-interacting protein kinase 6) (**Figure 8B**). Research has demonstrated that under drought stress, the expression of *GhCIPK6D1* and *GhCIPK6D3* is significantly upregulated, with *GhCIPK6D1* being associated with drought sensitivity in cotton, while *GhCIPK6D3* is linked to drought tolerance. Mechanistic studies reveal that the *GhCBL1A1-GhCIPK6D1* and *GhCBL2A1-GhCIPK6D3* complexes modulate stomatal aperture through regulation of K+ flux in guard cells, exhibiting both positive and negative regulatory effects on cotton’s drought tolerance (Sun et al., 2024). Overexpression of *GhCIPK6* significantly enhanced salt, drought, and ABA stress tolerance in transgenic *Arabidopsis*, indicating that *GhCIPK6* functions as a positive regulator in salt and drought stress responses and represents a potential candidate gene for improving stress tolerance through genetic manipulation (He et al., 2013). Our study identified *Ghi_A13G12271* (CIPK6) as the hub gene of the turquoise module, further confirming that CIPK6 is a key regulatory factor in the drought response of cotton.

Following activation via different signal transduction cascades, transcription factors modulate plant biological processes by binding to cis-regulatory elements and controlling expression of target genes (Nunavath et al., 2025). The transcription factor HvHSFA2e enhances drought and heat tolerance through coordinated upregulation of heat-responsive genes and modulation of ABA signaling and flavonoid biosynthesis pathways (Mishra et al., 2024). The Golden2-like transcription factor in maize enhances the drought tolerance of rice by promoting stomatal closure (Li et al., 2024a). Studies have shown that the C2H2 transcription factor can play an important regulatory role in the drought response of plants by mediating the transduction process of the ABA and other signaling pathways (Han et al., 2020). The TIFY transcription factor *ZmJAZ13* positively regulates the drought response of plants, and overexpressing this gene improves the drought tolerance of *Arabidopsis* (Zhang et al., 2025b). Additionally, Aux/IAA transcription factors contribute to drought resistance in Arabidopsis by modulating glucosinolate levels (Salehin et al., 2019). In addition, the MYB, bHLH, and GRAS transcription factor families can all participate in the transduction process of multiple signaling pathways by regulating the expression of various downstream genes, thus playing roles in the plant response to abiotic stresses (Lei et al., 2024; Liu et al., 2025; Zhang et al., 2025a). To further identify important genes associated with drought resistance, we conducted an analysis of highly connected transcription factors in the blue and turquoise modules. We found that the blue module contains seven classes of transcription factors, primarily including HSF, Golden2-like, SNF2, mTERF, bHLH, C2H2, and B3 (**Figure 8C**). The turquoise module comprises six classes of transcription factors, mainly including Tify, ARR-B, AUX/IAA, bHLH, Alfin-like, and LUG (**Figure 8D**). This suggests that these transcription factors may play significant roles in the drought resistance of cotton; however, their specific functions and regulatory mechanisms require further in-depth investigation.

## 5 Conclusion

Through comprehensive phenotyping and physiological assessments of ten cotton cultivars with varying drought tolerance, we identified the most resistant (64-22-3) and sensitive (A3) varieties. Transcriptome profiling revealed 7,351 DEGs in 64-22–3 and 5,009 DEGs in A3 under drought stress conditions (Drought treatment vs. well-watered controls). Parallel metabolomic analysis detected 169 and 173 significantly altered metabolites in 64-22–3 and A3, respectively. KEGG enrichment showed that both cultivars shared conserved drought-response pathways (secondary metabolite, flavonoid, and sesquiterpenoid/triterpenoid biosynthesis), while 64-22–3 specifically activated phenylpropanoid biosynthesis, linoleic acid metabolism, and glucosinolate biosynthesis pathways, likely enhancing drought tolerance. We performed WGCNA using 2,064 drought-specific DEGs and 20 unique DAMs from 64-22-3. This revealed significant correlations between 20 DAMs and two key modules: The blue module (hub gene: *Ghi_D06G05631*, a TOPLESS-related 1 protein) contained eight high-connectivity transcription factor families: HSF, Golden2-like, SNF2, mTERF, bHLH, C2H2, and B3. The Turquoise module (hub gene: *Ghi_D13G23691*, a CIPK6) comprised seven high-connectivity TF families: Tify, ARR-B, AUX/IAA, bHLH, Alfin-like, and LUG. This study provides further elucidation of the molecular response mechanisms underlying drought resistance in cotton, establishing a theoretical foundation for subsequent applied research and utilization of drought-resistant genes in cotton breeding.

## Data Availability

The raw transcriptome sequencing data generated in this study have been deposited in the Genome Sequence Archive (GSA) at the National Genomics Data Center, China National Center for Bioinformation/Beijing Institute of Genomics, Chinese Academy of Sciences, under accession number CRA024840. The metabolomics data are available in the OMIX repository (Metabolomics and Multi-Omics Data Archive) under accession number OMIX009884.
