# Correction: Comparative transcriptome and metabolome profiling unveil genotype-specific strategies for drought tolerance in cotton

**DOI:** 10.3389/fpls.2025.1699493

**Published:** 2025-10-21

**Authors:** Aixia Han, Wanwan Fu, Yunhao Liusui, Xingyue Zhong, Xin Zhang, Ziyu Wang, Yuanxin Li, Jingbo Zhang, Yanjun Guo

**Affiliations:** Xinjiang Key Laboratory of Special Species Conservation and Regulatory Biology, College of Life Science, Xinjiang Normal University, Urumqi, China

**Keywords:** cotton, drought-resistant varieties, transcriptome, metabolome, hub gene

In the published article, **Figure 2A** contains an error. Upon our verification, it was identified that the labels “A3” and “64-22-3” in **Figure 2A** were reversed: specifically, the image on the left should be labeled “64-22-3”, while the image on the right should be labeled “A3”. The corrected **Figure 2** is provided below.

**Figure 2:**
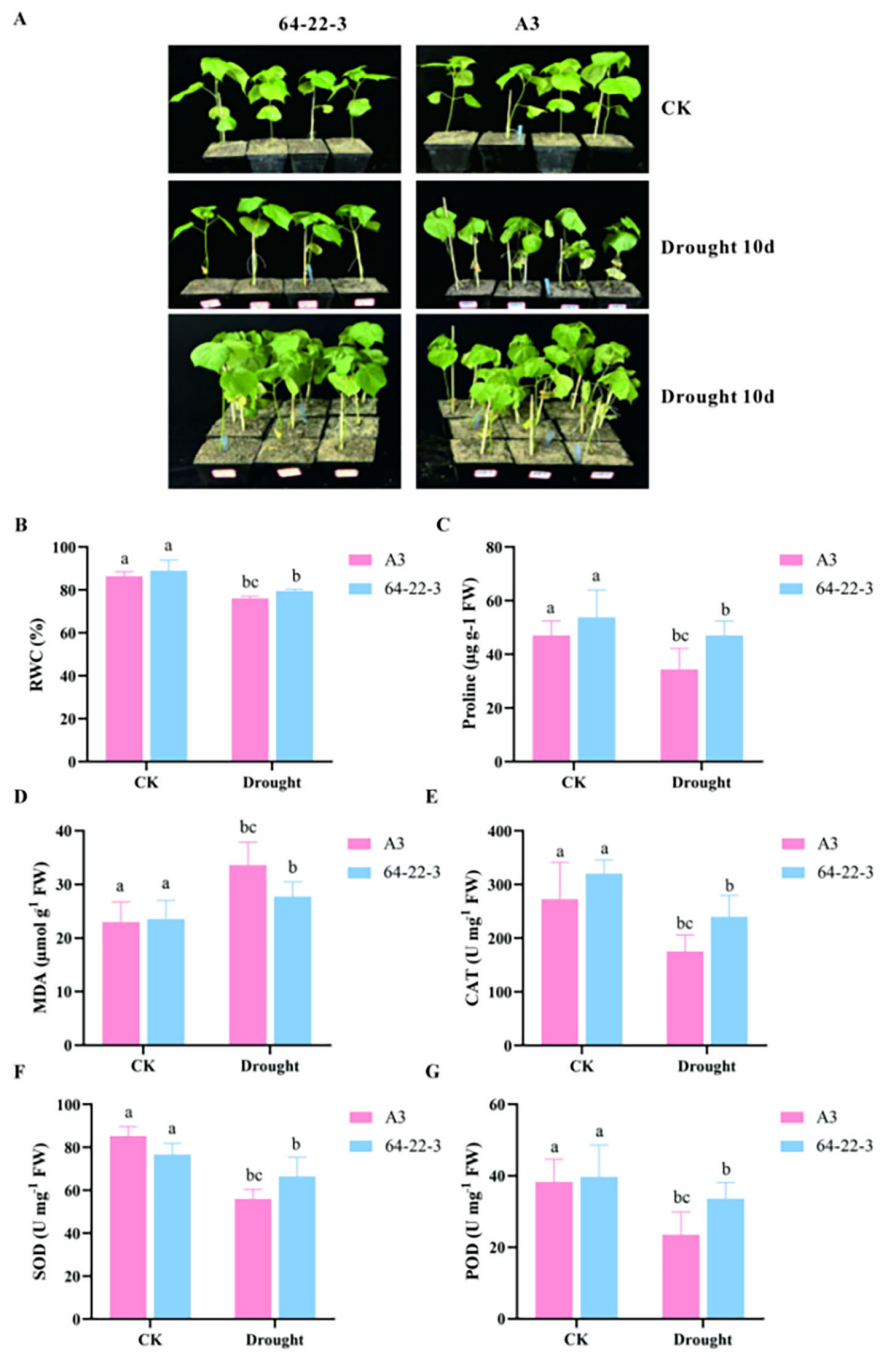
Phenotypic and physiological characterization of cotton under normal irrigation and drought conditions. Different lowercase letters indicate significant differences among treatment groups at p < 0.05 level. **(A)** Phenotypic comparison between normally irrigated and drought-stressed cotton plants; **(B)** Leaf relative water content (RWC, %) in control and drought-treated groups; **(C)** Proline content (Pro) under normal irrigation and drought stress; **(D)** Malondialdehyde content (MDA) in control and drought-exposed plants; **(E)** Catalase (CAT) activity in cotton under well-watered and drought-stress conditions. **(F)** Superoxide dismutase (SOD) content in cotton under well-watered and drought-stress conditions. **(G)** Peroxidase (POD) activity in cotton under well-watered and drought stress conditions.

The original version of this article has been updated.

