# Supplementary material for: Comparative transcriptome and metabolome profiling unveil genotype-specific strategies for drought tolerance in cotton
Source: Front Plant Sci. 2025 Jun 13;16:1610552. doi: 10.3389/fpls.2025.1610552 (PMC12202649; doi:10.3389/fpls.2025.1610552)
Supplement: Supplementary file 1 [file DataSheet1.docx]

Supplementary Material

# Supplementary Data

# Supplementary Figures and Tables

## Supplementary Figures


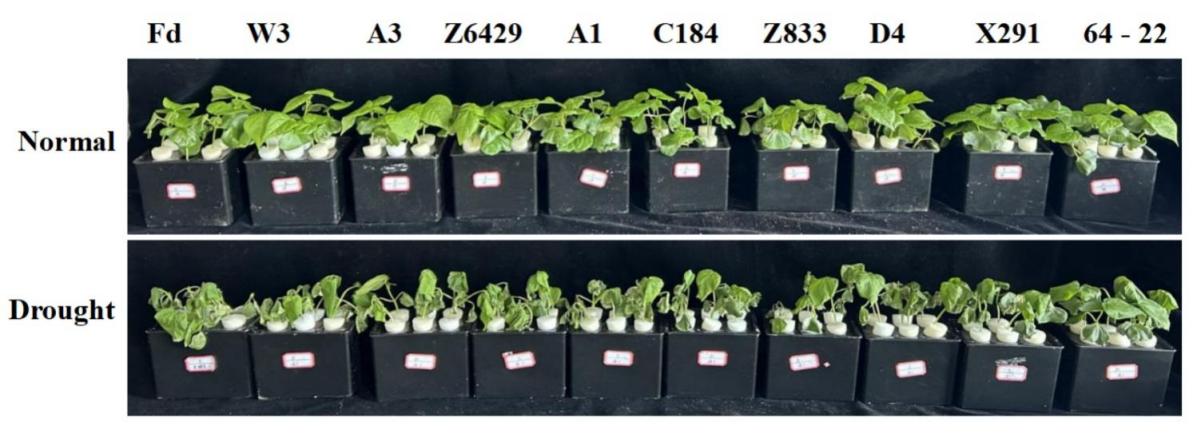


**Supplementary Figure 1.** Comparative analysis of the phenotypes of different cotton varieties under PEG simulated drought treatment conditions.


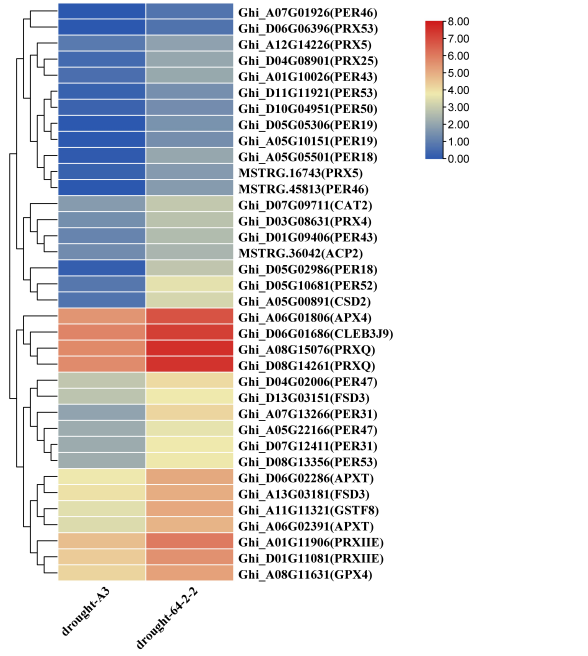


**Supplementary Figure 2.** Expression analysis of genes such as PRX, POD, SOD, APX and CAT in varieties 64-22-3 and A3 under drought stress conditions.


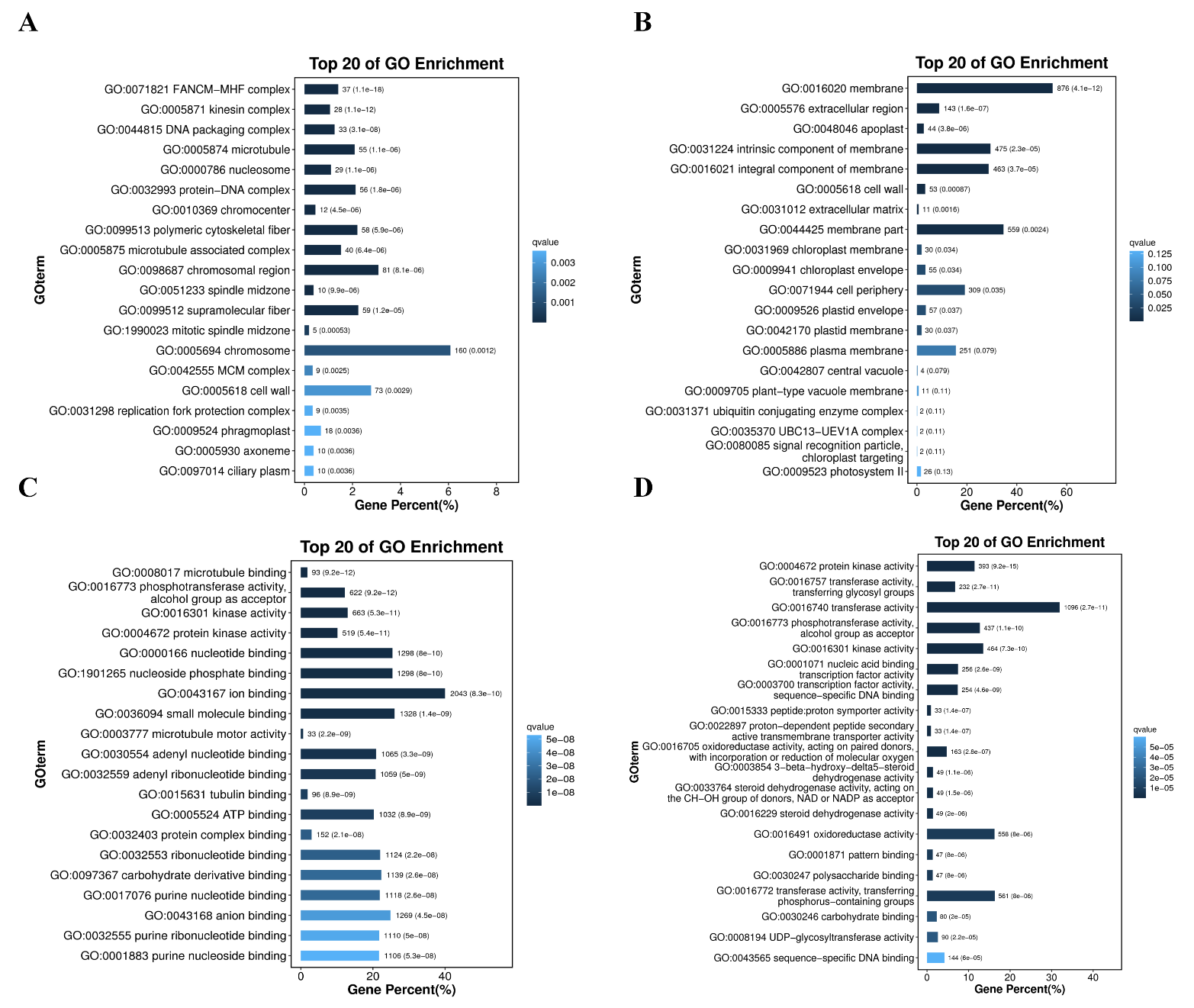


**Supplementary Figure 3.** GO enrichment analysis of DEGs between drought and normal watering groups. The x-axis "Gene Percentage" indicates the proportion of genes enriched in the corresponding GO terms, while the y-axis lists specific GO functional terms. All enriched terms were selected with a threshold of p-value ≤ 0.05. (A) GO enrichment analysis of cellular component for DEGs of 64-22-3. (B) GO enrichment analysis of cellular component for DEGs of A3. (C) GO enrichment analysis of molecular function for DEGs of 64-22-3. (D) GO enrichment analysis of molecular function for DEGs of A3.


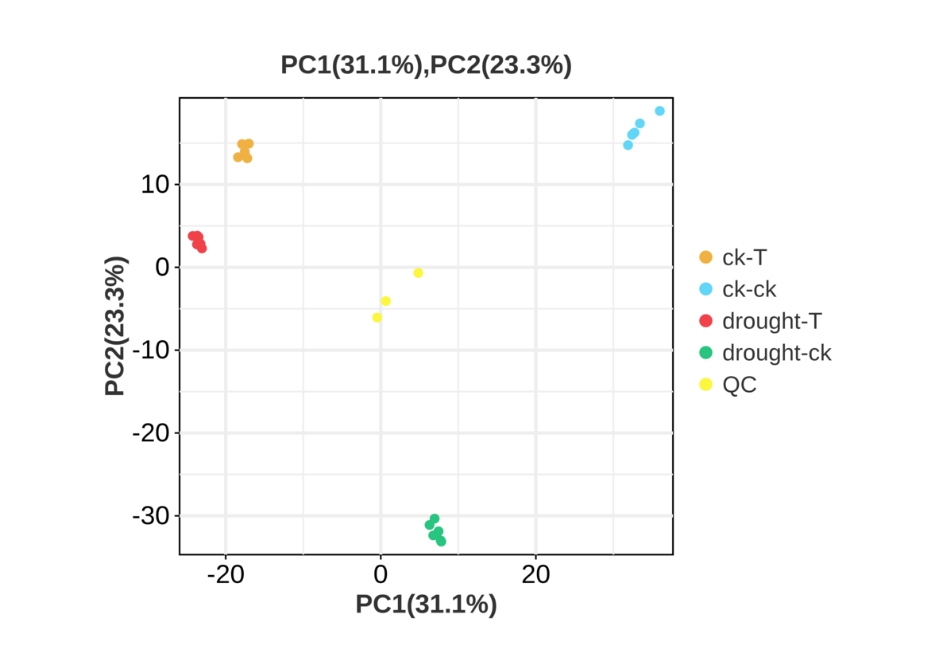


**Supplementary Figure 4.** Principal component analysis (PCA) of different metabolomic samples.


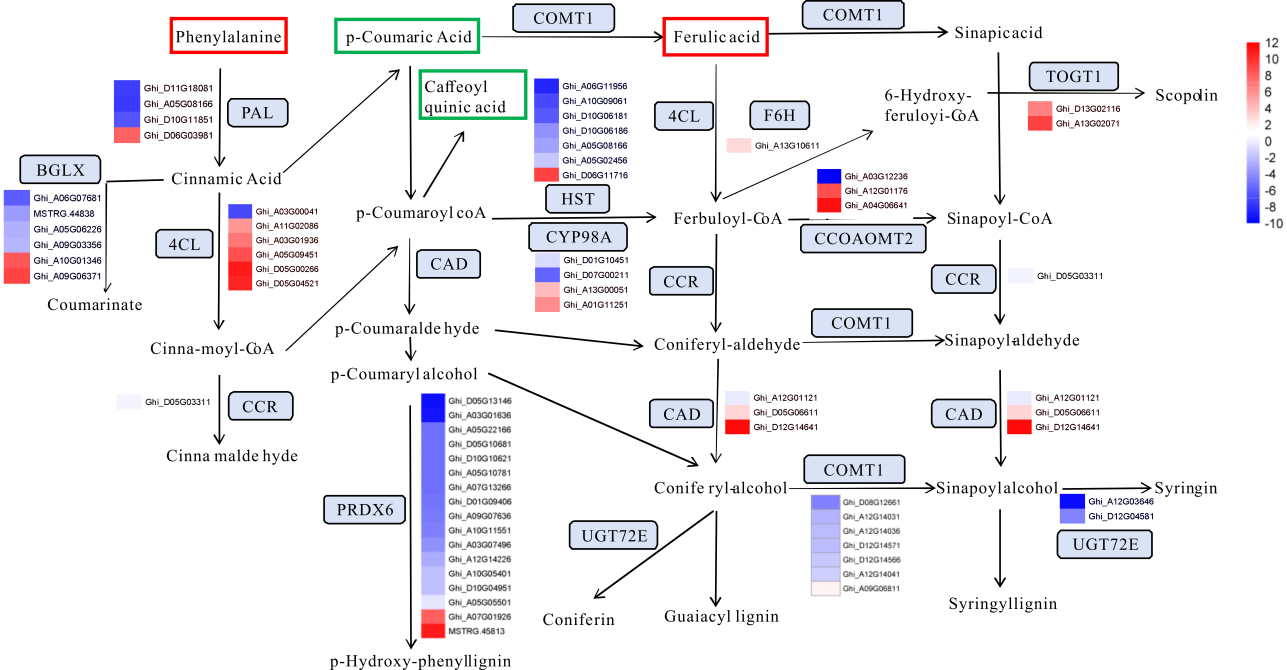


**Supplementary Figure 5.** Integrated network analysis reveals metabolite accumulation and associated gene expression patterns in the phenylpropanoid biosynthesis pathway under drought stress. The heatmap illustrates gene expression profiles (log2(FC) of Drought/CK in drought-tolerant cultivars). Red and green nodes represent up- and down-regulated metabolic components, respectively, in drought-treated samples compared to controls.


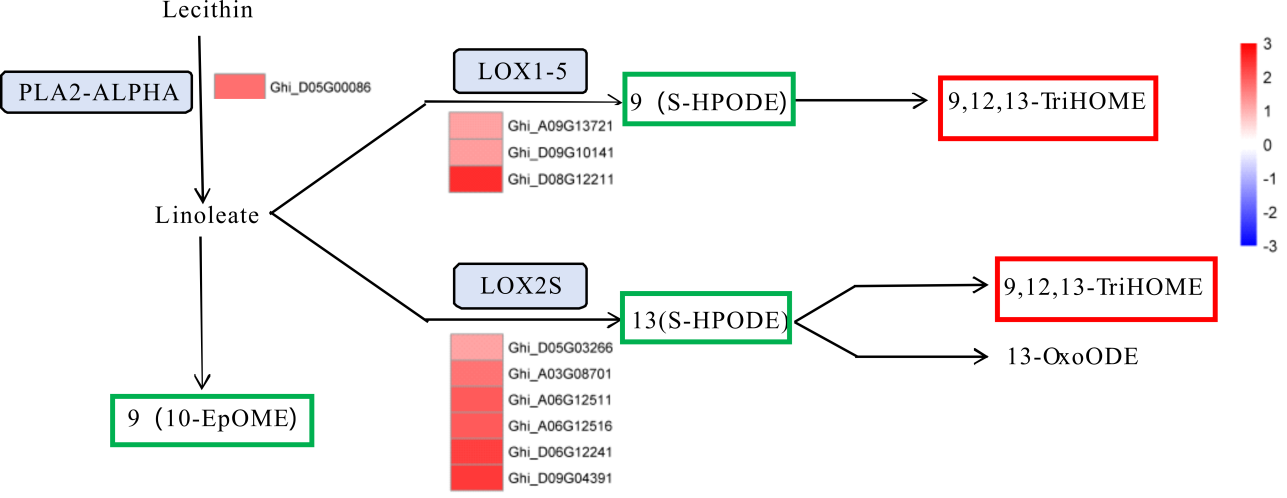


**Supplementary Figure 6.** Integrated network analysis reveals metabolite accumulation and associated gene expression patterns in the linoleic acid metabolism pathway under drought stress. The heatmap illustrates gene expression profiles (log2(FC) of Drought/CK in drought-tolerant cultivars). Red and green nodes represent up- and down-regulated metabolic components, respectively, in drought-treated samples compared to controls.


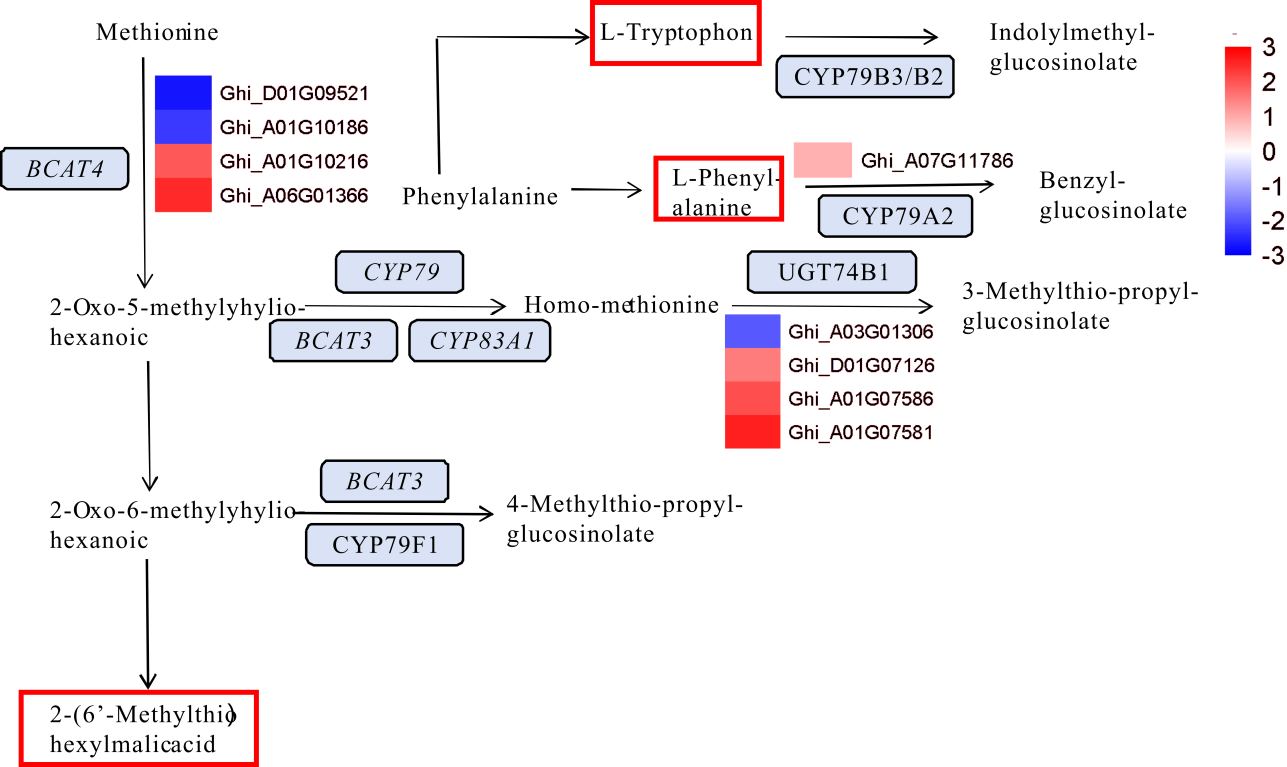


**Supplementary Figure 7.** Integrated network analysis reveals metabolite accumulation and associated gene expression patterns in the glucosinolate biosynthesis pathway under drought stress. The heatmap illustrates gene expression profiles (log2(FC) of Drought/CK in drought-tolerant cultivars). Red and green nodes represent up- and down-regulated metabolic components, respectively, in drought-treated samples compared to controls.


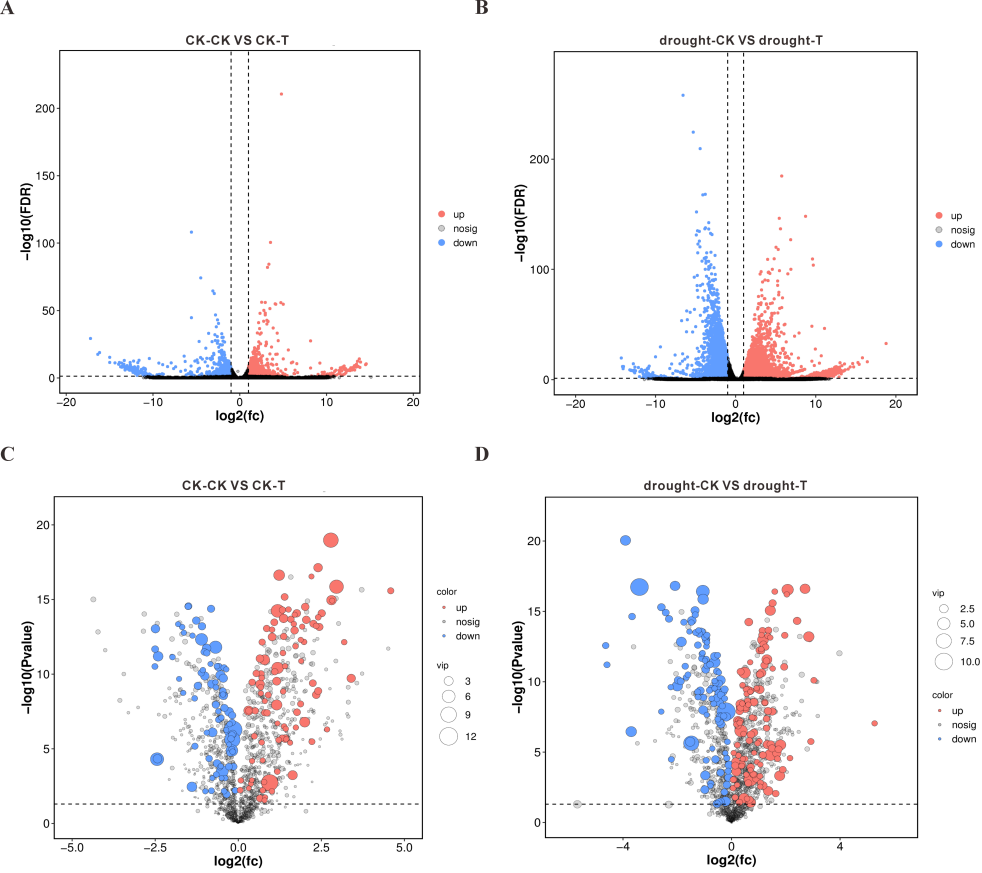


**Supplementary Figure 8.** Supplementary Figure 8. Volcano plots of DEGs and DAMs for various comparison groups. The x-axis represents log2(FC) of gene expression and metabolite abundance between comparison groups, while the y-axis shows -log10-transformed p-values . The dashed horizontal line represents the p-value threshold for screening DEGs and DAMs. Red dots represent significantly upregulated genes and metabolites, while blue dots denote significantly downregulated ones..(A) Volcano plot of DEGs between 64-22-3 and A3 under normal watering conditions. (B) Volcano plot of DEGs between 64-22-3 and A3 under drought conditions. (C) Volcano plot of DAMs between 64-22-3 and A3 under normal watering conditions. (D) Volcano plot of DAMs between 64-22-3 and A3 under drought conditions.


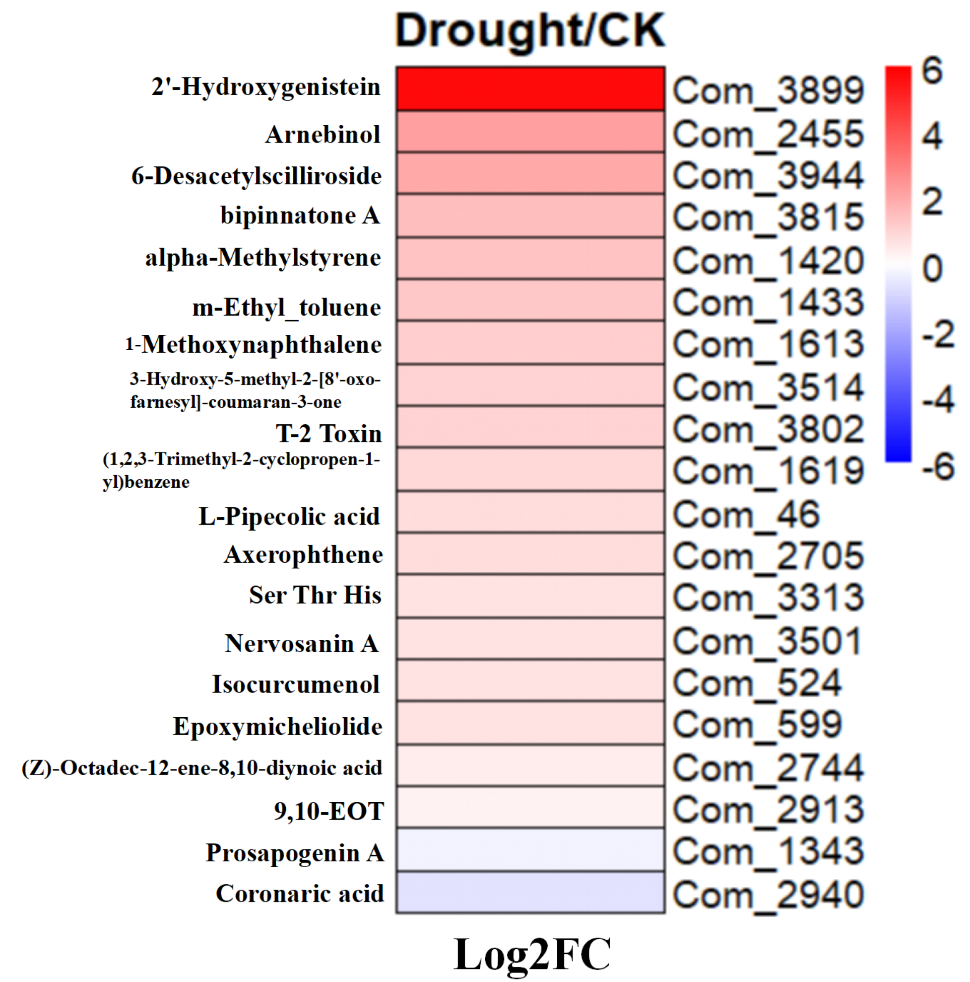


**Supplementary Figure 9.** Analysis of specific DAMs in the drought-resistant variety 64-22-3 under drought versus normal watering conditions.


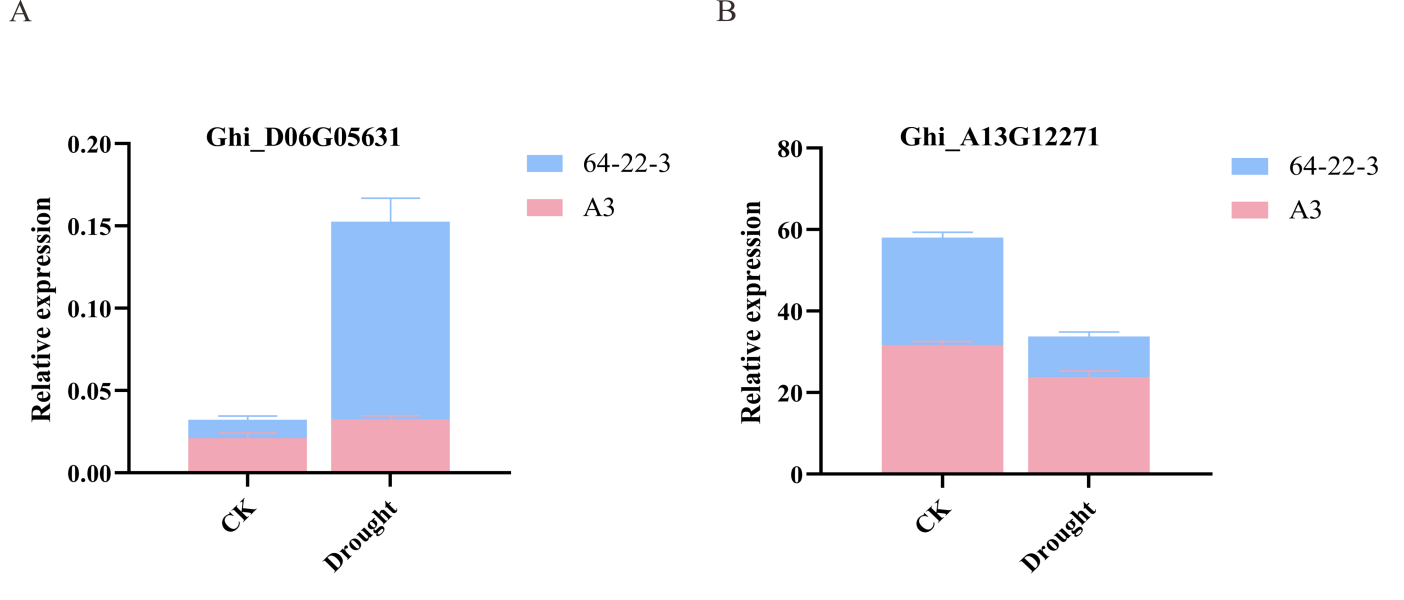


**Supplementary Figure 10.** Expression analysis of *Ghi_D06G05631* and *Ghi_A13G12271*.(A) qRT-PCR analysis of *Ghi_D06G05631* expression levels in cotton varieties 64-22-3 and A3. (B) qRT-PCR analysis of *Ghi_A13G12271* expression levels in cotton varieties 64-22-3 and A3.

## Supplementary Tables

| **RawData(bp)** | **BF_Q20(%)** | **BF_Q30(%)** | **BF_GC(%)** | **CleanData(bp)** | **AF_Q20(%)** | **AF_Q30(%)** | **AF_GC(%)** |
| --- | --- | --- | --- | --- | --- | --- | --- |
| **7150512000** | **97.5** | **93.5** | **43.57** | **6965116730** | **97.90** | **93.95** | **43.22** |
| **5500991400** | **97.97** | **94.3** | **43.92** | **5373203751** | **98.22** | **94.67** | **43.64** |
| **7947225900** | **98.14** | **94.76** | **43.96** | **7770971773** | **98.42** | **95.12** | **43.71** |
| **6606017400** | **98.09** | **94.61** | **44.07** | **6487190708** | **98.26** | **94.84** | **43.90** |
| **6525904800** | **98.30** | **95.09** | **43.97** | **6404650823** | **98.45** | **95.31** | **43.79** |
| **6274800900** | **97.96** | **94.29** | **44.17** | **6142662721** | **98.14** | **94.55** | **43.99** |
| **7981617600** | **97.93** | **94.43** | **44.13** | **7706598470** | **98.30** | **94.94** | **43.61** |
| **9032450700** | **97.56** | **93.64** | **43.80** | **8737538190** | **97.98** | **94.17** | **43.31** |
| **8732273700** | **97.75** | **93.88** | **43.51** | **8500587369** | **98.05** | **94.29** | **43.09** |
| **6528711000** | **98.11** | **94.68** | **43.80** | **6377132101** | **98.31** | **94.97** | **43.56** |
| **7616305800** | **98.24** | **94.93** | **42.76** | **7435506125** | **98.41** | **95.18** | **42.49** |
| **6406281900** | **98.08** | **94.67** | **44.33** | **6249831339** | **98.39** | **95.06** | **44.00** |

**Supplementary Tables 1.** Base Information Statistical Table. "Before filter (BF)" represents the base information of the samples before filtering; "After filter (AF)" represents the base information of the samples after filtering; "RawData (bp)" indicates the total number of bases in the data generated from sequencing (unit: bp); "CleanData (bp)" indicates the total number of bases in the high-quality data after filtering (unit: bp); "Q20 (%)" represents the number of bases with a sequencing base quality value reaching the Q20 level or above and the percentage of such bases in the RawData (or CleanData); "Q30 (%)" represents the number of bases with a sequencing base quality value reaching the Q30 level or above and the percentage of such bases in the RawData (or CleanData); "N (%)" represents the number of bases containing "N" in the single-end reads and the percentage of such bases in the RawData (or CleanData); "GC (%)" represents the GC ratio of the bases in the sequences before (or after) filtering.
